# Maladaptive spinal plasticity opposes spinal learning and recovery in spinal cord injury

**DOI:** 10.3389/fphys.2012.00399

**Published:** 2012-10-10

**Authors:** Adam R. Ferguson, J. Russell Huie, Eric D. Crown, Kyle M. Baumbauer, Michelle A. Hook, Sandra M. Garraway, Kuan H. Lee, Kevin C. Hoy, James W. Grau

**Affiliations:** ^1^Department of Neurological Surgery, Brain and Spinal Injury Center, University of California San FranciscoSan Francisco, CA, USA; ^2^Abbott LaboratoriesChicago, IL, USA; ^3^Department of Neurobiology, University of Pittsburgh School of MedicinePittsburgh, PA, USA; ^4^Cellular and Behavioral Neuroscience, Department of Psychology, Texas A&M UniversityCollege Station, TX, USA

**Keywords:** pain, nociception, plasticity, spinal cord injury, spinal cord learning, recovery of function

## Abstract

Synaptic plasticity within the spinal cord has great potential to facilitate recovery of function after spinal cord injury (SCI). Spinal plasticity can be induced in an activity-dependent manner even without input from the brain after complete SCI. A mechanistic basis for these effects is provided by research demonstrating that spinal synapses have many of the same plasticity mechanisms that are known to underlie learning and memory in the brain. In addition, the lumbar spinal cord can sustain several forms of learning and memory, including limb-position training. However, not all spinal plasticity promotes recovery of function. Central sensitization of nociceptive (pain) pathways in the spinal cord may emerge in response to various noxious inputs, demonstrating that plasticity within the spinal cord may contribute to maladaptive pain states. In this review we discuss interactions between adaptive and maladaptive forms of activity-dependent plasticity in the spinal cord below the level of SCI. The literature demonstrates that activity-dependent plasticity within the spinal cord must be carefully tuned to promote adaptive spinal training. Prior work from our group has shown that stimulation that is delivered in a limb position-dependent manner or on a fixed interval can induce adaptive plasticity that promotes future spinal cord learning and reduces nociceptive hyper-reactivity. On the other hand, stimulation that is delivered in an unsynchronized fashion, such as randomized electrical stimulation or peripheral skin injuries, can generate maladaptive spinal plasticity that undermines future spinal cord learning, reduces recovery of locomotor function, and promotes nociceptive hyper-reactivity after SCI. We review these basic phenomena, how these findings relate to the broader spinal plasticity literature, discuss the cellular and molecular mechanisms, and finally discuss implications of these and other findings for improved rehabilitative therapies after SCI.

## Introduction

Research into spinal plasticity over the past 50 years has shown that neurons within the spinal cord gray matter have a remarkable degree of plasticity, and in recent years we have seen a surge of interest in this field (Raineteau and Schwab, 2001; Edgerton et al., 2004; Cai et al., 2006; Grau et al., 2006). Spinal synapses can be strengthened or weakened in response to external stimulation, demonstrating the basic properties required for use-dependent learning and memory. This capacity is of great importance after spinal cord injury (SCI). Prior work has shown that peripheral injury or inflammation can induce chronic neuropathic pain states and that this outcome is due, in part, to sensitization of nociceptive pathways within the spinal cord (for review see Woolf and Salter, 2000; Ji et al., 2003). Aspects of this process have been shown to be mechanistically analogous to brain-dependent learning and memory, and thus represents a lasting form of maladaptive spinal plasticity. Work from our team over the past 15 years has built upon this foundation, focusing on the ways in which central sensitization impacts and informs both adaptive and maladaptive forms of spinal learning and memory (Ferguson et al., 2006; Grau et al., 2006; Hook et al., 2008; Huie et al., 2012a,b). Our work suggests that exposure to uncontrollable/unpredictable peripheral stimulation induces a central sensitization-like state that inhibits adaptive spinal learning and undermines recovery of locomotor function after spinal contusion injury.

The present review discusses the features of spinal cord plasticity with a specific emphasis on protecting against maladaptive plasticity in nociceptive systems and promoting adaptive forms of spinal plasticity for rehabilitation after SCI. We will first review the cellular and physiological evidence for use-dependent spinal cord plasticity and draw parallels to brain-dependent plasticity. We then review behavioral evidence for learning and memory within the spinal cord and discuss how specific changes in stimulation parameters can tip the balance between adaptive and maladaptive outcomes. Finally, we discuss how noxious input below the level of SCI may induce a similar nociceptive plasticity that undermines recovery, and the potential application of appropriate spinal cord training to overcome these maladaptive effects to restore function after SCI.

## Cellular and electrophysiological evidence for synaptic plasticity in the spinal cord

Research of spinal plasticity over the past 50 years has eroded the perception of the spinal cord as a simple conduit of neural information. We now know that that the spinal cord is capable of supporting a number of forms of plasticity, yet spinal plasticity remains understudied in comparison to the field of brain plasticity. We will begin this review by highlighting some of the major findings that reshaped our view of the spinal cord, in order to provide the reader with a contextual background for our later discussions of the ways in which spinal plasticity is modulated.

Synaptic changes in the spinal cord have been often studied in the context of spinal nociceptive plasticity. In the early 1980s Clifford Woolf demonstrated that tissue injury to the lateral hind-paw produces cutaneous hypersensitivity to light tactile (von Frey) stimulation both on the ipsilateral and contralateral hind-paw (Woolf, 1983). This suggested that post-injury pain hypersensitivity was due, in part, to an increase in excitability of spinal cord neurons. This increase in spinal activity, known as “central sensitization,” reflects a form of neuroplasticity in the spinal cord (Woolf and Salter, 2000).

In many ways central sensitization involves changes in the spinal cord gray matter that mimic hippocampal-mediated activity-dependent plasticity. In the hippocampus, tetanic stimulation of afferent pathways can increase responsiveness of subsequent post-synaptic potentials, a phenomenon known as long-term potentiation (LTP) (Bliss and Lomo, 1973). LTP is widely believed to be a synaptic mechanism for learning and memory in the CNS. Early pharmacological evidence suggested that both hippocampal LTP and hippocampal-dependent learning tasks are blocked by antagonists to the glutamate NMDA receptor (Collingridge and Bliss, 1987). Other lines of evidence suggested that electrophysiologically overdriving (saturating) LTP prevented both later LTP and spatial learning (McNaughton et al., 1986; Moser et al., 1998). Recent data have shown that spatial learning experience produces LTP in the hippocampus that is detectable both electrophysiologically (as increases post-synaptic currents) and biochemically as an increase in phosphorylation of the glutamate AMPA receptor and trafficking of AMPA receptors to synapses (Whitlock et al., 2006). These AMPA receptor changes are thought to be fundamental to LTP and other forms of plasticity at excitatory synapses in the CNS (Malinow and Malenka, 2002).

Many of the characteristics of hippocampal LTP have been identified in the spinal cord, providing a potential cellular mechanism for central sensitization. For example, tetanic stimulation of primary nociceptive afferents (C-fibers) has the capacity to increase post-synaptic responses in the superficial spinal lamina, a phenomenon known as “wind-up” (Mendell and Wall, 1965; Mendell, 1966). In addition, like hippocampal LTP, central sensitization can be blocked with NMDA receptor antagonists (Woolf, 1983; Woolf and Thompson, 1991; Dougherty et al., 1992), providing a strong pharmacological link between nociceptive sensitization and LTP. This apparent common mechanism between changes in nociception and LTP (which has been suggested as a substrate for learning and memory) has led led to the idea that nociceptive sensitization may act as a form of ‘pain memory’ (Woolf and Costigan, 1999; Ji et al., 2003). Work by Sandkuhler and colleagues have provided direct evidence that nociceptive stimuli can produce spinal LTP (Liu and Sandkuhler, 1995; Sandkuhler and Liu, 1998). In addition, this team has recently found that high doses of short-acting opioids can reverse spinal pain memory, as measured by losses of hyper-reactivity, reduced spinal LTP, and reduced phosphorylation of glutamate AMPA receptors (Drdla-Schutting et al., 2012). Together, these data provide strong evidence that activity-dependent plasticity in pain pathways reflects a form of learning and memory within the spinal cord.

Spinal glia have also been implicated in spinal LTP. Once believed to simply provide structural support, the capacity for glial cells to affect glutamatergic signaling through the release of a host of neuromodulators has led researchers to assess the importance of glia in CNS plasticity (Muller, 1992; Allen and Barres, 2005). Using high-frequency stimulation of the sciatic nerve to induce LTP of C-fiber-evoked field potentials in the dorsal horn, Ma and Zhao demonstrated that glial activity is required for this effect, as spinal treatment with the glial metabolic inhibitor fluorocitrate blocked the induction of spinal LTP (Ma and Zhao, 2002). As spinal pain memory is believed to be encoded by a LTP-like effect in the dorsal horn, this finding illustrates the essential role of glia in modulating spinal plasticity. A similar effect has been demonstrated behaviorally as well. Watkins et al. induced pain hyper-reactivity in rats using a peripheral injection of the common irritant formalin. They found that if glial activity was inhibited using fluorocitrate prior to formalin administration, the induction of this hyper-reactivity was blocked (Watkins et al., 1997). Others have investigated the key glial products that mediate neuromodulation of nociceptive plasticity in the spinal cord, and have shown a number of these products to be involved, including nitric oxide, prostaglandins, and the cytokines IL-1b, IL-6, and tumor necrosis factor alpha (TNFα). TNFα in particular has been shown to enhance spinal LTP in rats with neuropathic pain (Liu et al., 2007). In both hippocampal culture and *in vivo* spinal cord, TNFα has been shown to increase trafficking of AMPA receptors to synaptic sites, providing a potential mechanism for TNFα-induced increases in spinal LTP (Beattie et al., 2002; Ferguson et al., 2008b; Choi et al., 2010). Recent work aimed at elucidating the role of spinal glia and TNFα in maladaptive forms of spinal nociceptive plasticity is discussed later in this review.

Similarly, metabotropic glutamate receptors (mGluRs) modulate spinal plasticity within pain pathways by altering the plasticity of the ionotropic NMDA and AMPA receptors (Mills et al., 2002). In particular, the group I mGluRs (mGluR1 and mGluR5) have been shown to enhance ionotropic receptor-dependent central nociceptive plasticity in the spinal cord (Fisher and Coderre, 1996a,b). These systems have also been implicated in brain-dependent plasticity as well as multiple forms of spinal plasticity. We will return to a discussion of mGluRs in the “cellular and molecular mechanisms” section of this review.

In summary, the spinal cord is capable of supporting memory for prior noxious experience that manifests behaviorally, pharmacologically, and electrophysiologically. This spinal memory depends on mechanisms similar to learning and memory in the higher CNS, including induction and expression of LTP at spinal synapses. Spinal LTP is mediated by at least some of the same receptor pathways as in the brain, providing further evidence of a common mechanism of plasticity. Notably, the expression of LTP in spinal pain pathways has been shown to contribute to central sensitization in nociceptive systems, providing a mechanism for some maladaptive neuropathic pain states.

## Spinal cord learning and memory

Plasticity within the spinal cord is not limited to maladaptive plasticity within nociceptive pathways. The spinal cord also demonstrates several forms of adaptive motor plasticity. In the following section, we will move beyond spinal nociceptive pathways to investigate how spinal plasticity in motor pathways can induce robust behavioral changes, and how these changes can be used as outcome measures in a simple model of learning in the spinal cord.

Inducing adaptive plasticity in spinal motor systems can have profound effects on locomotor behavior. For example, following complete thoracic transection, the lumbar spinal cord can regain the capacity to sustain weight-supported stepping with extensive step training (Lovely et al., 1986; Barbeau and Rossignol, 1987; de Leon et al., 1998; Harkema et al., 2011). The capacity for locomotor re-training after SCI is thought to be possible because the lumbar spinal cord contains central neural networks that control reciprocal activity of extensor and flexor efferents during locomotion (Grillner, 1975; Grillner and Zangger, 1979). These “central pattern generators” in the lumbar cord can be tuned by generating a specific pattern of afferent input during physical rehabilitation training, thereby promoting recovery of function (Dietz and Harkema, 2004; Prochazka and Yakovenko, 2007; Edgerton et al., 2008).

However the specific learning capacities of the spinal cord that underlie this recovery of function remain a topic of intensive study. Work from the field of neurobiology of learning and memory has revealed that the isolated spinal cord can support simple forms of motor learning. There is well-documented evidence that spinal neurons can sustain single stimulus learning (habituation/sensitization), stimulus association (Pavlovian conditioning), and response-outcome (instrumental) learning (Sherrington, 1906; Thompson and Spencer, 1966; Fitzgerald and Thompson, 1967; Grau et al., 1998).

Early demonstrations of habituation and sensitization in the spinal cord provided fundamental evidence that the spinal cord could learn from repeated activity, and demonstrated a form of spinal memory that manifested behaviorally. Repeated exposure to a stimulus was found to decrease (habituate) a spinally mediated flexion response. This habituation was not due to adaptation at sensory receptors in the periphery or to a change at the neuromuscular junction, suggesting that the memory for stimulus training history resided in spinal interneuronal synapses (Sherrington, 1906; Prosser and Hunter, 1936; Thompson and Spencer, 1966). In contrast to habituation, which occurred with moderate stimuli, exposure to a single strong stimulus had the capacity to increase subsequent responsiveness in a process known as “sensitization”. Groves and Thompson (1970) went on to characterize different interneuronal pools that were responsible for habituation and sensitization, providing one of the early neurobiological theories of activity-dependent plasticity in the spinal cord.

Another line of work revealed that spinal neurons were capable of encoding relationships between different stimuli, a hallmark of Pavlovian (classical) conditioning. For example, the isolated spinal cord was shown to be capable of associating weak thigh stimulation (conditioned stimulus; CS) with strong plantar stimulation of the foot (unconditioned stimulus, US). With repeated CS–US pairings the thigh stimulation came to modulate the flexion withdrawal reflex, a type of Pavlovian conditioning known as “pairing-specific enhanced sensitization” (Fitzgerald and Thompson, 1967; Groves et al., 1969). A similar form of Pavlovian conditioning had been demonstrated in the aquatic mollusk, Aplysia, in classic work in the Kandel laboratory (Carew et al., 1981). Together these data suggest that not all forms of learning and memory require the brain.

Much of our recent work has focused on response-outcome (instrumental) training of the spinal cord, with the goal of uncovering the basic principles that dictate spinal cord learning. The translational goal of this work is to provide basic knowledge that can help improve training-based rehabilitative therapies after SCI. Our spinal training model is based on the Horridge paradigm originally developed in headless insects and then later adapted to spinalized mammals to study learning and memory within the spinal cord (Horridge, 1962; Buerger and Fennessy, 1970; Grau et al., 1998). Horridge found that after experimentally instituting a relationship between leg position and electrical stimulation of an ankle flexor, insects could learn to hold the hind limb in a flexed position. This acquired leg flexion emerged after repeated trials, demonstrating an acquisition curve that resembles brain-dependent escape learning. To rule out the possibility that the change in flexion response reflected a peripheral mechanism such as muscle tetanus, Horridge used a clever design that involved testing insects in “master/yoked” pairs. One insect, the master, received leg stimulation only when the leg was extended. The other rat served as a yoked control that received passive leg stimulation whenever the master received stimulation. Although both rats received equal stimulation, only the master insect learned to hold the leg in a flexed position, suggesting that the acquired flexion response was not due to a simple unconditioned effect of stimulation, but rather depended on the response-outcome contingency between leg position (response) and stimulation (outcome).

**Figure 1:**
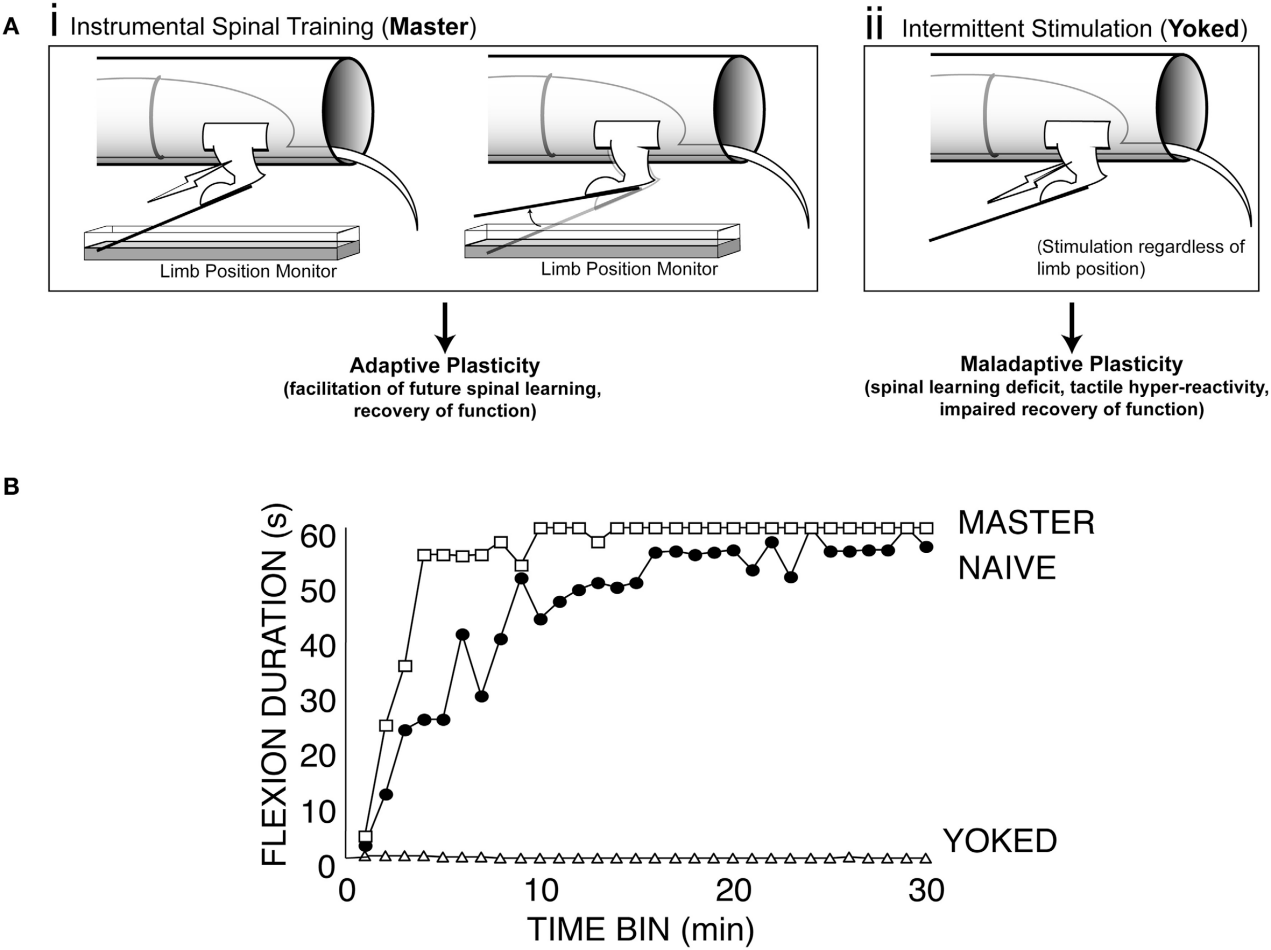
**Effects of instrumental vs. uncontrollable nociceptive stimulation on spinal function. (A)** Modes of stimulation. (i) Instrumental spinal training. For Master rats, electrical stimulation is delivered to the tibialis anterior muscle when the hindlimb is unflexed, and terminated when the hindlimb is flexed. Over a 30 min training session, master rats learn to keep the hindlimb flexed to reduce stimulus exposure (spinal learning). This spinal training promotes future adaptive plasticity. (ii) Uncontrollable stimulation. The yoked rats receive stimulation whenever the master does, regardless of their hindlimb position. **(B)** Exposure to this uncontrollable (yoked) stimulation causes a learning deficit that is evident when these rats are later tested with the spinal learning assay. Spinal learning is assessed by monitoring the ability of spinally transected rats to maintain the hindlimb in a flexed position as manifested by increasing average response duration in each 1 min time bin for 30 min test during instrumental training. Modified from Grau et al. (1998).

Master/yoked training of the hind limb after spinal transection in rats revealed that the mammalian spinal cord is also sensitive to response-outcome relationships (Figure 1A; Buerger and Fennessy, 1970). Building upon this foundation, our group has shown that this example of spinal cord plasticity meets the behavioral criteria (Grau et al., 2006) for instrumental learning and has a lasting effect on spinal function that impacts clinically relevant phenomena (e.g., Grau et al., 1998, 2004; Ferguson et al., 2006; Hook et al., 2008). Over the past 14 years we, and others (Jindrich et al., 2009), have shown that by imposing a relationship between leg position and stimulation of the tibialis anterior muscle (master condition/controllable stimulation) we can produce beneficial effects that improve future spinal cord training (Figure 1B, master; Grau et al., 1998). In this sense the spinal cord shows memory for training history and can re-learn the flexion response more rapidly after instrumental training (Figure 1B, master vs. naïve).

However, not all spinal memory for stimulus training is adaptive. Rats that are given stimulation that is independent of leg position (yoked condition/uncontrollable stimulation) show persistent deficits in future learning when tested with response-contingent stimulation. This spinal learning deficit appears to reflect a long-term form of maladaptive spinal plasticity that endures for at least 48 h in completely transected rats (Figure 1B, yoked; Grau et al., 1998; Crown et al., 2002b). Thus, using this master/yoked learning paradigm has allowed us to observe and manipulate the expression of both adaptive and maladaptive forms of plasticity in the spinal cord.

The training-dependent effects of stimulus controllability on future spinal learning can be interpreted as “plasticity of plasticity” or “metaplasticity,” that regulates the capacity for future learning in a bidirectional manner (Abraham and Bear, 1996; Crown et al., 2002a,b; Grau et al., 2006; Ferguson et al., 2008b). On the one hand, controllable stimulation produces a positive and adaptive form of plasticity that promotes future spinal learning and limits the development of nociceptive plasticity. The benefits of controllable stimulation are discussed in detail in Grau et al. (2012) within this same issue. On the other hand, uncontrollable stimulation produces a lasting, maladaptive effect that undermines the capacity for future spinal learning. A large body of work from our group and others has focused on the stimulus parameters that are critical to this effect, the neurobiological mechanisms that mediate this form of maladaptive plasticity, and how it may relate to the development of central sensitization. Perhaps most importantly, we have worked to uncover how such an effect impacts recovery of function following SCI.

The remaining sections of this review will delve into these critical issues. The general theme is that uncontrollable/yoked stimulation produces a maladaptive form of spinal metaplasticity that is associated with impaired spinal learning, reduced recovery of function after SCI, and nociceptive hyper-reactivity (Ferguson et al., 2006; Hook et al., 2008). The findings above suggest that the specific patterning of peripheral stimulation exerts exquisite control over the nature of activity-dependent plasticity that develops in the spinal cord. In sections that follow we will review the environmental conditions that determine whether stimulation impacts spinal function in an adaptive or maladaptive manner and the underlying neurobiological mechanisms.

## Stimulation parameters for modulating spinal learning

The capacity for adaptive spinal cord learning is thought to contribute to functional recovery after SCI (Edgerton et al., 2001; Grau et al., 2006). However, the specific stimulus conditions that promote effective spinal cord learning are not fully understood. Similarly, those conditions that lead to a spinal learning deficit require elucidation. Given that SCI is likely to be accompanied by peripheral noxious input from other concomitant injuries (as well as noxious input as a result of secondary injury processes), a more complete understanding of the stimulus parameters that may undermine adaptive spinal plasticity is essential. This section will highlight findings that have shed light on the conditions under which maladaptive plasticity may be induced.

After careful study of yoked animals, Crown and Grau (2001) developed a computer program that emulated the shock schedule produced by master rats during the early phase of training. This enabled the experimental evaluation of the effects of uncontrollable stimulation in a single “virtually yoked” rat to explore the parameters that disable future learning ability. The program delivers 80 ms, 1.5 mA AC current (60 Hz) stimulation on a randomized variable interstimulus interval that ranges from 0.2 to 3.8 s (mean interval = 2 s). Through a series of parameteric studies Crown et al. (2002a) found that delivering as little as 6 min of uncontrollable stimulation to either the leg or the tail after complete spinal transection produces lasting (>48 h) impairments in spinal learning (Crown et al., 2002a; Joynes et al., 2003). Later work went on to show that the same stimulation procedure produced long-term (>6 weeks) impairments in recovery of function after contusive SCI (Grau et al., 2004). As these effects are seen in both transection and contusion injuries the findings suggest that intermittent, uncontrollable stimulation has a generalized negative effect on adaptive spinal plasticity.

The finding that intermittent uncontrollable stimulation to the tail produced the same disruption of adaptive motor learning as stimulation to the leg provided important support for the idea that these maladaptive changes are centrally mediated in the spinal cord. But the question remained, what is it about these particular stimulation parameters that drives such robust inhibition of spinal learning and recovery? Is it the uncontrollability, the number of shocks presented, the unpredictability, or some combination thereof that is essential to this phenomenon?

The lasting differences in learning capacity between master/yoked pairs suggested that controllability is an essential characteristic. The differential effects of controllable and uncontrollable stimulation on spinal plasticity can be invoked even when the total amount of stimulation is equalized using master/yoked training protocol. In master/yoked training, rats are run in pairs where one rat (master) is given response-contingent stimulation and the other rat (yoked) receives stimulation whenever the master does. Even though the two rats receive stimulation at the same time, only master rats show instrumental learning. But is it necessary that stimulation pulses be separated at all to induce the yoked learning failures? Is it the total amount of shock delivered that predicts the induction of a spinal learning deficit, or the discontinuous nature of shock administration? Delivering 80 ms stimulation at an average of 2 s intervals over 6 min produces a total of approximately 180 shocks, and a total stimulation time of approximately 15 s. Thus, to test whether these temporal gaps are necessary, we applied a continuous 1.5 mA AC (60 Hz) shock for 15 s to one group, while another group received a continuous AC shock for the entire 6-min session. A third group received the typical intermittent stimulation schedule, and a control group received no stimulation. All rats were then subsequently tested for instrumental learning. As expected, the rats that received no stimulation were able to learn, and those that received intermittent stimulation failed. Interestingly, both groups that received continuous stimulation were able to learn, suggesting that the long-term effect of shock stimulation depends upon whether the pulses are separated by a temporal gap. Moreover, presenting a continuous tailshock while rats received intermittent legshock negated the adverse effect of intermittent stimulation, suggesting that continuous stimulation induces an opponent process that counters the induction of the learning deficit (Crown et al., 2002a).

**Figure 2:**
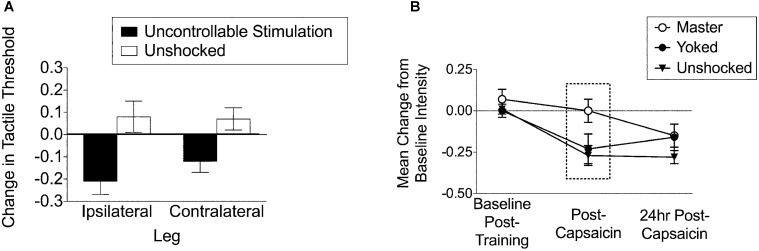
**Training history alters nociceptive hyper-reactivity. (A)** Uncontrollable stimulation (6 min, 80 ms stimuli, variable inter-stimulus interval: 0.8–3.2 s, 1.5 mA AC) to the leg produces tactile hyper-reactivity on both the ipsilateral and contralateral leg. The same stimulation pattern undermines spinal cord learning on both leg for over 24 h and impairs recovery of locomotor function for over 6 weeks (adapted from Ferguson et al., 2006). **(B)** Master and yoked training have differential effects on central sensitization to intradermal capsaicin. Rats that have been trained with controllable stimulation (Master) have decreased tactile responsiveness immediately post-capsaicin injection [adapted from Hook et al. (2008)]. Similar effects are reported with central sensitization by intradermal formalin in Ferguson et al., 2012 within this same issue.

Further work revealed that continuous and intermittent stimulation have divergent effects on nociceptive reacitivity. Exposure to continuous shock reduces reactivity (antinociception) to a noxious thermal stimulus applied to the tail (tail-flick test; Crown et al., 2002a). In contrast, exposure to intermittent shock has no effect on thermal reactivity (Crown et al., 2002a), but enhances reactivity to tactile stimulation. Rats were given uncontrollable intermittent stimulation to the hindleg and then tested for tactile reactivity on the ipsilateral and contralateral leg using calibrated von Frey hairs. The results indicated that uncontrollable stimulation to the leg produces a bilateral tactile hyper-reactivity, whereas controllable (instrumental) stimulation has the opposite effect, mitigating central sensitization (Figure 2; Ferguson et al., 2006; Hook et al., 2008; Huie et al., 2012b). Thus, it appears that one key feature that may differentiate the effects of intermittent stimulation from other stimulation schedules is the induction of nociceptive sensitization.

**Figure 3:**
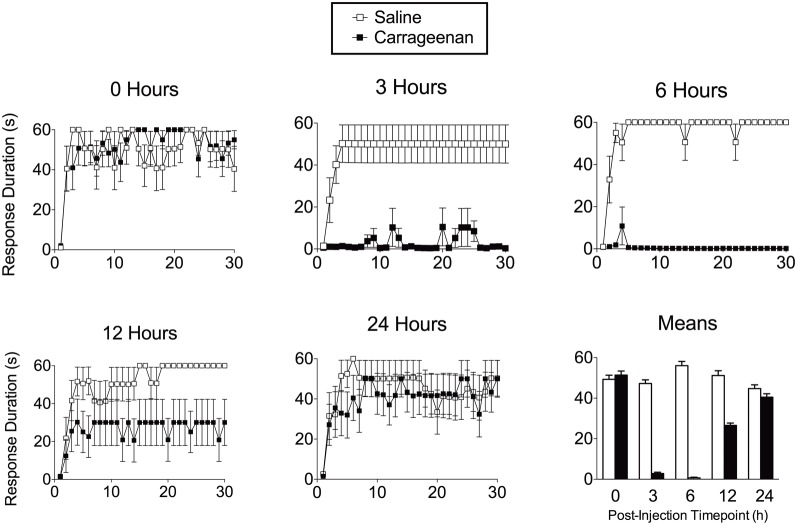
**Peripheral inflammation with intradermal carrageenan produces transient impairment in spinal cord learning on the contralateral leg.** The timecourse of these learning deficits mimic the known timecourse for carrageenan-induced central sensitization. Adapted from Ferguson et al. (2006).

The enhanced reactivity to tactile stimulation observed after intermittent shock resembled the mechanical allodynia observed after peripheral inflammation and injury (Ji et al., 2003; Hook et al., 2008). Given this, we hypothesized that the maladaptive effect of uncontrollable stimulation may be linked to the induction of a state akin to central sensitization. If so, it would be expected that the spinal learning deficit should be induced not only within the confines of specific electrical stimulation parameters, but by using more generalized nociceptive activators. We discovered that intradermal injection of the inflammatory agent carrageenan, an agent often reported in the pain literature to induce central sensitization, also produced a marked inhibition of spinal learning (Figure 3; Ferguson et al., 2006). This effect was time-dependent with the most dramatic loss of spinal learning at 3–6 h, and recovery of learning by 24 h after injection. This time-course precisely mirrors the established time-course for central sensitization by carrageenan (Hargreaves et al., 1988; Zhang et al., 2003). Similar effects have subsequently been observed with other peripheral nociceptive stimuli including intradermal capsaicin and formalin (Hook et al., 2008). These findings suggest that intermittent stimulation undermines adaptive spinal modifications by inducing nociceptive plasticity that is akin to central sensitization.

**Figure 4:**
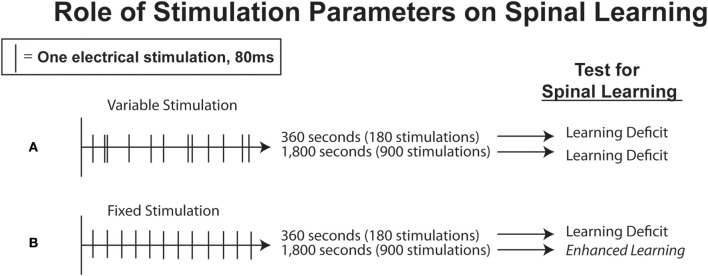
**Stimulation parameters affecting spinal learning. (A)** If nociceptive electrical stimulation is delivered to the tail in a randomized, intermittent fashion, rats will fail to learn when later tested with controllable stimulation to one hindlimb. This deficit is exhibited after either short (360 s) or long (1,800 s) trains of intermittent, randomized stimulation. **(B)** If stimulation is administered to the tail with a fixed interval between stimulations over the course of 360 s, rats will also later fail to learn, but if the train of fixed stimulation is extended to 1800 s rats then exhibit enhanced learning when later tested. Adapted from Baumbauer et al. (2008).

In all of the experiments reviewed above, intermittent shock was presented in a variable manner using a program that emulated the temporal distribution of shocks produced by a master rat. Does this temporal variable matter, or would intermittent shock undermine spinal plasticity independent of whether it is presented in variable or regular (fixed spaced) manner? Baumbauer et al. (2008) explored this issue and found that spacing does not matter when rats are given just 6 min of stimulation (approximately 180 shocks); both fixed and variably spaced shock impaired subsequent learning (Figure 4; Baumbauer et al., 2008). In contrast, when rats were exposed to an extended series of shocks (1800 given over 30 min), only variable shock induced a learning deficit. Introducing temporal regularity (predictability) not only eliminated the learning deficit, it induced a protective effect analogous to instrumental control that both prevented, and reversed, the learning deficit (Baumbauer et al., 2009). The protective effect of fixed spaced shock lasted 24 h was mediated by a protein synthesis-dependent process, and involved a form of NMDAR-mediated plasticity (Baumbauer et al., 2009). These observations suggest that both instrumental control and temporal predictability can negate the adverse consequences of intermittent stimulation. Variable shock may lead to a central sensitization-like state independent of shock number because this pattern of stimulation emulates the erratic pattern of neural activity observed in C-fibers (Sandkuhler, 2007). Further, intermittency may be an essential feature because repeated shock onsets are required to drive an electrophysiological response over extended periods; a continuous stream of shock pulses may lead to a form of physiological habituation that reduces C-fiber induced activity (Groves and Thompson, 1970).

In summary, several studies have demonstrated that the patterning of peripheral stimulation after SCI can have an enormous effect on whether adaptive or maladaptive plasticity emerges in the lumbar spinal cord. Response-contingent (master), predictable nociceptive stimulation promotes future spinal cord learning whereas unpredictable, intermittent nociceptive stimulation undermines future spinal learning and generates central sensitization. Therefore, discovering the biological mechanisms for these stimulus-induced forms of spinal plasticity has implications for both pain modulation and recovery of function after SCI.

## Cellular and molecular mechanisms dictating spinal learning

The preceding sections have outlined the fundamental features of spinal cord learning, and highlighted the importance of stimulus patterning in tipping the balance between adaptive and maladaptive forms of spinal plasticity. In the present section we will discuss the cellular and molecular mechanisms dictating spinal learning potential. The purpose of this line of research has been to discover biological mechanisms that modulate spinal learning, with the goal of developing therapeutic approaches to promote rehabilitation and recovery of function after SCI (Grau et al., 2006). Because of its rapid-throughput nature (30 min), the instrumental training assay has provided a powerful tool to identify a number of neuropharmacological targets for improving adaptive spinal cord function. In general, this work has focused on either promoting adaptive plasticity to improve future spinal learning, or preventing maladaptive plasticity to reverse learning deficits and limit nociceptive plasticity. This section will first briefly consider the mechanisms that mediate spinal learning, and will then focus on the neurobiological pathways that have been implicated in the maladaptive effect of uncontrollable, intermittent nociceptive stimulation.

Adaptive spinal cord learning in the Horridge paradigm is blocked by intrathecal lidocaine, indicating that spinal neuronal activity is required for the learned flexion response (Crown et al., 2002b). In addition, intrathecal antagonists to glutamate NMDA and AMPA receptors have each been independently shown to block acquisition of the flexion learning (Joynes et al., 2004; Hoy et al., 2012). These findings mimic what is observed in visuo-spatial learning paradigms, indicating that spinal cord learning in the Horridge paradigm depends on biological mechanisms similar to hippocampal-dependent learning in the brain (Whitlock et al., 2006). This suggests that mechanisms that enhance hippocampal-dependent learning have potential to enhance spinal cord training as well. Gomez-Pinilla et al. (2007) tested the relationship between spinal training and several known molecular biomarkers of CNS plasticity. Quantitative RT-PCR revealed that master rats have an increase in spinal mRNA levels compared to naïve controls for several pro-plasticity biomarkers including brain-derived neurotrophic factor (BDNF), calcium/calmodulin activated kinase II (CaMKII), cAMP response element binding protein (CREB), and the pre-synaptic terminal protein synapsin I. Moreover, the degree of mRNA correlated highly with performance on the spinal learning task. In contrast, yoked animals demonstrated significant decreases in spinal levels of BDNF, CaMKII, and CREB relative to naïve animals. Together these data suggest that pro-plasticity markers may play a role in spinal cord learning.

BDNF appears to be particularly important for spinal learning. Intrathecal BDNF supplementation can promote spinal cord learning when task difficulty is increased beyond a level that naïve rats can normally perform (Gomez-Pinilla et al., 2007). In contrast the BDNF inhibitor TrkB-IgG can block the adaptive benefits of prior training reducing master animals to the performance level of untrained rats. This suggests that BDNF, a well-established modulator of synaptic plasticity in the brain, also has beneficial effects on adaptive spinal plasticity that promotes future spinal learning (reviewed in McAllister et al., 1999; Fritsch et al., 2010). In contrast, the learning deficit following yoked stimulation involves a down regulation of BDNF, suggesting that bidirectional modulation of synaptic plasticity mechanisms dictate spinal learning potential. A similar role for BDNF in promoting adaptive spinal plasticity has also recently been shown to translate to a model of locomotor recovery. Boyce et al. demonstrated that in rats with a complete transection at the T10 thoracic spinal cord segment, treatment with adeno-associated virus expressing BDNF was able to induce weight-supported hindlimb stepping without the assistance of step-training (Boyce et al., 2012).

**Figure 5:**
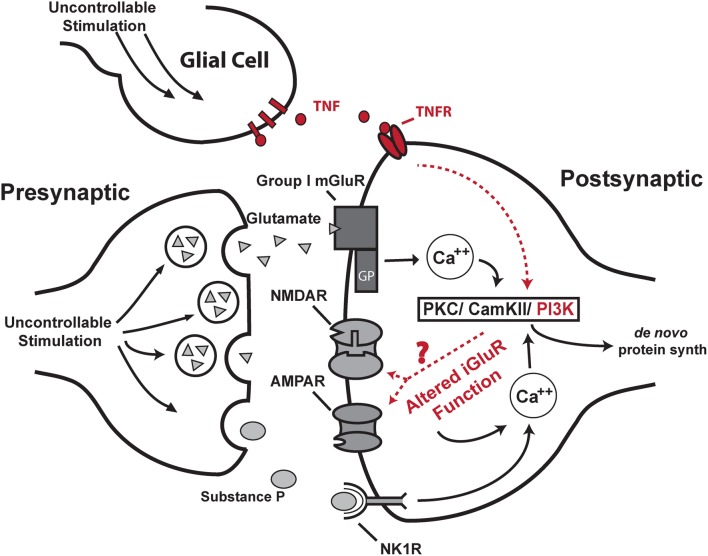
**Proposed neurobiological model for maladaptive spinal plasticity.** The uncontrollable stimulation-induced spinal learning deficit requires the activation of group I metabotropic glutamate receptors and the substance P receptor NK1R which liberate intracellular calcium (Baumbauer et al., 2008; Ferguson et al., 2008a). This in turn activates downstream protein kinases PKC and CamKII, (Ferguson et al., 2006, 2008a; Baumbauer et al., 2007). These kinases are known to produce long-term alterations in ionotropic glutamate receptor (iGluR) function; however, the specific role of this signaling cascade in spinal learning remains an open question (“?”; dashed lines). Altered iGluR activation is known to further increase post-synaptic calcium levels through the NMDA receptor channel and calcium-permeable AMPA receptors, which may provide a mechanism for altered associative learning in the spinal cord. Increases in intracellular calcium can induce further protein kinase activity and *de novo* protein synthesis, all of which have all been shown necessary for the development of the stimulation-induced spinal learning deficit (Patton et al., 2004; Baumbauer et al., 2006; Huie et al., 2012a). Well-characterized features are shown in black. Areas of ongoing study are shown in red. Adapted from Ferguson et al. (2008a).

Further work has indicated that uncontrollable/unpredictable intermittent nociceptive stimulation engages a maladaptive form of plasticity that involves its own signature of cellular molecular changes. Initial studies indicated that intrathecal lidocaine (Joynes et al., 2003) and protein synthesis inhibitors (Patton et al., 2004; Baumbauer et al., 2006) protect learning potential when delivered prior to intermittent nociceptive stimulation. This suggested that spinal cord circuitry actively encodes the maladaptive stimulation patterns through a form of activity-dependent CNS plasticity. Indeed, intrathecal delivery of an NMDA receptor antagonist (at doses that are known to block central sensitization of nociceptive systems) prior to intermittent nociceptive stimulation protected spinal learning potential (Ferguson et al., 2006). This observation led to the hypothesis that central sensitization undermines spinal cord learning potential by engaging a generalized hyper-excitability that prevents adaptive spinal learning (Ferguson et al., 2006). Several converging lines of molecular and cellular evidence have lent support for this concept. The key cellular and molecular mechanisms associated with both long-term spinal learning deficits and central sensitization are depicted in Figure 5. Intermittent nociceptive stimulation has been found to engage several glutamate receptor systems, as well as the proinflammatory cytokine TNFα and substance P (Baumbauer et al., 2007; Huie et al., 2012a). All of these systems have been implicated in central nociceptive processing as well (Sandkuhler and Gruber-Schoffnegger, 2012).

One of the conundrums outlined above is that both adaptive learning and maladaptive plasticity could be blocked by NMDA receptor antagonists (Joynes et al., 2004; Ferguson et al., 2006). This suggested that NMDA receptors serve as executors of spinal plasticity in the spinal learning preparation. The NMDA receptor in spinal motoneurons has been shown to be highly mobile, and can induce glutamatergic plasticity by rapidly trafficking from extrasynaptic to synaptic sites in response to changes in glutamatergic input (Shanthanelson et al., 2009). Interestingly, Shanthanelson et al. showed that recovery of NMDA-mediated excitatory tone was more pronounced at monosynaptic input sites coming from segmental dorsal roots, as opposed to central inputs coming from the ventrolateral funiculus. This finding suggests that NMDA-mediated plasticity is highly regulated by, and highly susceptible to, peripheral input. As such, differences in the timing and pattern of peripheral input (such as those seen in controllable vs. uncontrollable stimulation) may induce differential NMDA receptor trafficking and activation, leading to divergent forms of spinal plasticity.

Critical systems for switching between maladaptive and adaptive plasticity may also lie upstream of NMDA receptor changes. One of the well-established neuropharmacological modulators of NMDA receptor plasticity is group I mGluRs. The group I mGluRs consist of two receptor subtypes (mGluR1 and mGluR5) that are known to modulate plasticity in the CNS through down-stream effects on iGluRs, including NMDA and AMPARs (Fisher et al., 2002; Fundytus et al., 2002). In addition activation of group I mGluRs in the spinal cord increases NMDA-dependent central sensitization after intradermal formalin (Coderre and Melzack, 1992), and central pain after SCI (Mills et al., 2002).

Therefore, mGluRs are a good candidate for a central role in dictating spinal metaplasticity in the spinal learning paradigm. We tested this hypothesis using a combination of pharmacological, biochemical, and behavioral methods. We found that mGluR1 and mGluR5 antagonists protect spinal learning potential in the face of uncontrollable stimulation. On the other hand the group I mGluR agonist DHPG can substitute for uncontrollable stimulation to produce long-term impairments in spinal cord learning (Ferguson et al., 2008b). Group I mGluRs were the first receptor system identified to be both necessary and sufficient for inducing persistent maladaptive alterations in spinal learning potential. Maladaptive mGluR effects depend on downstream activation of protein kinase C (PKC), a molecule that has been implicated in hippocampal plasticity (Akers et al., 1986; Malinow and Malenka, 2002; Ferguson et al., 2008b). Activation of mGluRs and PKC has previously been implicated in maladaptive plasticity within spinal pain pathways, once again suggesting an interaction between spinal learning deficits and nociceptive systems (Munro et al., 1994; Young et al., 1995; Ferguson et al., 2008b).

One of the major downstream effects of mGluR and PKC activation is post-translational modification of the ionotropic AMPA receptor (Ugolini et al., 1997; Xiao et al., 2001). AMPA receptors are thought to mediate the majority of rapid excitatory neurotransmission in the CNS, and their post-translational modification can have major effects on synaptic plasticity. For example, phosphorylation of AMPARs can increase their open channel time, resulting in enhanced post-synaptic currents and LTP (Derkach et al., 1999; Lee et al., 2000). In addition rapid trafficking of AMPA receptors to the plasma membrane can have a major effect on CNS plasticity (Malinow and Malenka, 2002). In the spinal cord, AMPAR phosphorylation and trafficking to synapses have been implicated in central sensitization and other forms of maladaptive plasticity (Galan et al., 2004; Ferguson et al., 2008a; Drdla-Schutting et al., 2012).

**Figure 6:**
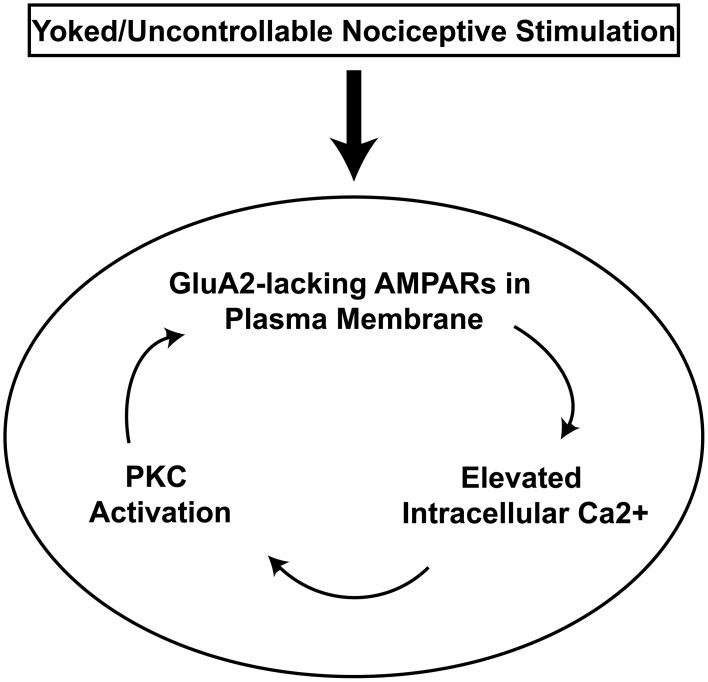
**AMPA receptor positive feedback-loop hypothesis for spinal metaplasticity.** Spinal AMPARs are hypothesized to rapidly traffic to the plasma membrane in response to uncontrollable nociceptive stimulation in the periphery (box). Because GluA2-lacking AMPARs function as calcium ionophores, their localization to the membrane generates a cascade of increased intracellular calcium followed by activation of intracellular calcium detectors such as PKC that can then produce further increases in trafficking of AMPARs to the plasma membrane. This feedback loop (oval) of increasing AMPA receptor numbers may account for the observed pattern of saturation of spinal learning plasticity, spinal learning deficits, and nociceptive hyper-responsiveness that is observed after yoked/uncontrollable stimulation training.

Pharmacological evidence has recently linked AMPAR over-activation to spinal learning deficits after intermittent nociceptive stimulation. Intrathecal delivery of a general AMPA receptor antagonist prevents induction of learning deficits by intermittent nociceptive stimulation (Hoy et al., 2012). However, this protective effect can only be observed at 24 h post-drug because general AMPAR antagonism reduces performance of the learned flexion response in the Horridge paradigm. A specific antagonist to AMPA receptors lacking the GluA2 subunit, however, selectively reverses the spinal learning deficit with no measurable side-effect on performance of the flexion response (Huie et al., 2012a). This is particularly relevant from a plasticity perspective because the GluA2 subunit renders AMPA receptors impermeable to Ca++ and GluA2-lacking receptors increase intracellular Ca++ levels which then, in turn, can activate a series of intracellular changes that can lead to a feedback loop of ever increasing excitatory plasticity and ultimately cellular dysfunction (Figure 6). Therefore, by engaging GluA2-lacking AMPARs intermittent nociceptive stimulation may produce lasting maladaptive plasticity that is characterized by spinal hyper-activity and reduced capacity for future spinal cord learning. However, the specific mechanisms by which intermittent nociceptive stimulation engages AMPAR receptors remains a topic of intensive ongoing study.

One prevailing hypothesis is that proinflammatory cytokine TNFα contributes to maladaptive spinal plasticity mediated by AMPA receptors. Prior work has shown that the glial TNFα increases trafficking of GluA2-lacking AMPA receptors to the neuronal plasma membrane, thereby increasing synaptic receptor levels (Beattie et al., 2002; Stellwagen et al., 2005; Stellwagen and Malenka, 2006; Leonoudakis et al., 2008). A series of studies have linked TNFα modulation of AMPARs to altered synaptic plasticity in the cortex (Leonoudakis et al., 2004; Jia et al., 2007), hippocampus (Furukawa and Mattson, 1998; Beattie et al., 2002; Ogoshi et al., 2005), and spinal cord (Hermann et al., 2001; Ferguson et al., 2008a). In addition, TNFα induced AMPAR trafficking has recently been implicated in maladaptive plasticity in pain pathways (Choi et al., 2010). We have recently undertaken a series of experiments to test whether TNFα plays a role in maladaptive plasticity in the spinal learning paradigm (Huie et al., 2012a). Results suggest that glial TNFα does indeed play a critical role in undermining spinal learning potential (Huie et al., 2012a). Intrathecal delivery of TNFα was found to be sufficient to induce a spinal learning deficit for at least 24 h. In addition, uncontrollable stimulation produces TNF release in the spinal cord, providing a link to stimulation-induced maladaptive plasticity. The maladaptive effects of uncontrollable stimulation and TNFα are reversible with glial inhibitors and TNFα sequestering agents, providing strong evidence that glial TNF plays a central role in modulation of synaptic mechanisms of spinal cord learning. We also found that specifically blocking GluA2-lacking AMPARs protected against both the TNF- and stimulation-induced learning deficits (Vichaya et al., 2009; Huie et al., 2012a). The specific mechanisms of these effects remain a topic of intensive study (Garraway et al., 2012; Stuck et al., 2012). However, several converging lines of evidence suggest that TNFα may play an important role in tipping spinal plasticity toward a maladaptive form that undermines future spinal cord training and promotes nociception. This crucial role for TNFα in mediating maladaptive spinal plasticity caudal to a complete transection may also provide insight into the recovery of function after spinal contusion injury. TNF mRNA is significantly increased following spinal contusion, peaking within hours, but remaining elevated for days (Wang et al., 1996). Likewise, others have shown that TNF protein signaling at the site of injury may not peak until 2 days after injury and remain elevated up to one week (Gorio et al., 2005). Given our findings that increased TNFa expression can undermine adaptive plasticity, it is possible TNFa may also contribute to limiting behavioral recovery after contusion injury by altering plasticity.

Taken together, these findings provide strong evidence that the maladaptive effect of intermittent stimulation on spinal learning is a distinct form of plasticity that shares many mechanistic similarities with central sensitization. Ongoing research into these molecular mechanisms may have great potential for improving rehabilitation therapies. The final section explores how the findings from the spinal learning work has been extended to spinal contusion injury, and how our work on maladaptive spinal plasticity may provide insights toward improved rehabilitation after injury.

## Implications for rehabilitation after spinal cord injury

We have shown how uncontrollable nociceptive stimulation can undermine adaptive spinal modifications below a SCI. Although much of this work was done in an isolated spinal system, the implications of these findings on recovery of function after incomplete SCI are profound. In the present section we will first show how our instrumental spinal training paradigm can be applied to a more general spinal injury model, and then discuss the broader implications of spinal training as means to overcome maladaptive plasticity, and promote functional recovery.

**Figure 7:**
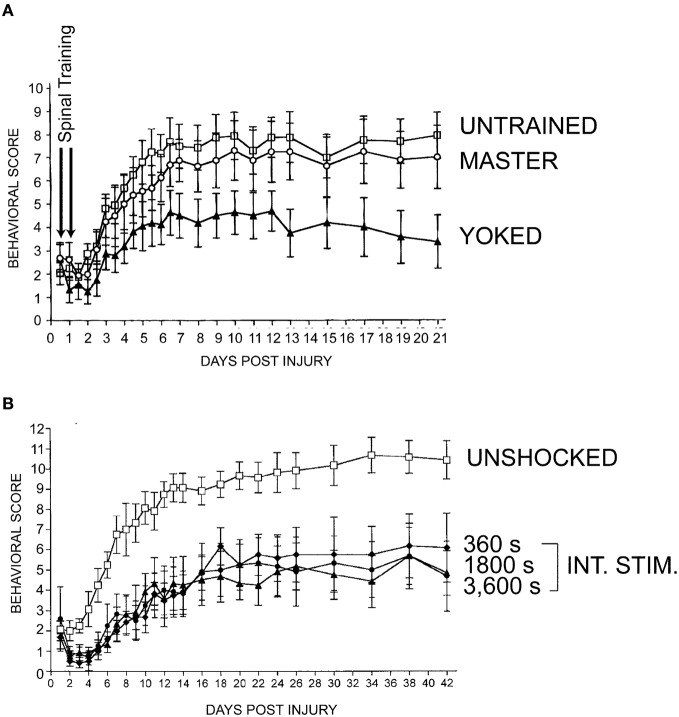
**The spinal learning deficit is associated with other forms of maladaptive plasticity such as impaired recovery of function after SCI. (A)** Rats were given 2 days of spinal training in the acute phase after a thoracic contusive SCI delivered with the NYU/MASCIS impactor (Gruner, 1992). **(B)** Varying amounts (in seconds, s) of intermittent stimulation (INT. STIM.) were administered to the tail one day following contusive SCI. Data show as little as 6 min (360 s) of intermittent stimulation to the tail is sufficient to undermine recovery of function. Y-axes represent a 12-point modified version of the Basso, Beattie, Bresnahan (BBB) locomotor scale, over 3 weeks **(A)** or 6 weeks **(B**) (Basso et al., 1995; Ferguson et al., 2004). Modified from Grau et al. (2004).

Knowing that the spinal cord is capable of exhibiting varying types of spinal plasticity in response to subtle changes in stimulation, we reasoned that spinal instrumental training could provide a window into how these divergent forms of spinal plasticity may dictate functional recovery after SCI. To test the relationship between instrumental training and recovery of function after SCI we evaluated the long-term effects of acute master/yoked training in a T12 contusion model of SCI (Grau et al., 2004). We delivered two 30 min sessions of master/yoked training to rats 24 and 48 h post-contusion injury and then monitored recovery of locomotor function over 42 days using the Basso, Beattie, Bresnahan (BBB; Basso et al., 1995) locomotor scale with behavioral raters who were blind to instrumental training condition. Yoked training produced persistent impairments in recovery of locomotor function (Figure 7A) as well as measures of sensory and autonomic recovery (Grau et al., 2004). Surprisingly, master rats did not show significant impairments. The yoked stimulation effect was further replicated, as just a single 6-min bout of uncontrollable stimulation to the tail, given 1 day post-injury, produced a deficit in locomotor recovery that was still apparent 6 weeks after injury (Figure 7B).

Such a finding illustrates just how vulnerable the spinal cord is to nociceptive input after injury, and how important the spinal cord is in processing nociceptive signals. This has clear translational relevance to human SCI, given that acute nociceptive input is a common feature of polytraumatic automobile accidents, the most common etiology of human SCI (Marino et al., 1999). Pain is a prevalent feature in SCI, affecting between 65–85% of the patient population (Siddall et al., 2003; Siddall, 2009). Although the mechanisms for neuropathic pain after SCI are not fully understood, the work reviewed in the present paper strongly suggests that co-morbid peripheral injuries may play a role in dictating both functional recovery and nociceptive sensitization.

One of the most clinically difficult forms of pain following SCI involves pain below the injury site. This phenomenon, known as dysesthetic pain syndrome, may represent an example of plasticity within spinal pain systems following SCI (Davidoff et al., 1987; Yezierski, 1996; Bennett et al., 2000; Hains et al., 2001; Bruce et al., 2002; Mills et al., 2002; Finnerup et al., 2003; Andersen et al., 2004; Carlton et al., 2009; Gwak et al., 2009; Hulsebosch et al., 2009; Leem et al., 2010). It has been argued that dysesthesia involves dysregulation of descending pain inhibitory mechanisms that affect spinal nociceptive circuitry. Two observations provide support for this. First, dysesthetic pain syndrome is most prevalent when there is damage specific to the spinal and mesencephalic trajectories of the ascending pain transmission pathways (Beric et al., 1988). These pathways are thought to be involved in activating segmental and descending pain inhibitory mechanisms. In contrast, most studies have shown that complete injury to the anterolateral ascending pain transmission pathways does not produce dysesthetic pain (Beric, 1993; Yezierski, 1996; Finnerup et al., 2003; Wasner and Brock, 2008). Second, there is evidence that many of the descending fibers damaged by SCI have a net inhibitory effect in uninjured subjects. Under non-pathological conditions, nociceptive plasticity in the spinal cord is tightly controlled by descending modulation from the brain, preventing spontaneous emergence of maladaptive pain syndromes. Following SCI there is significant alteration of reflexes below the lesion (Hubscher and Johnson, 2000; Grau and Patterson (eds.), 2001). Nociceptive reflexes demonstrate increased excitability accompanied by a loss of GABAergic inhibition (Zhang et al., 1994; Yezierski, 1996), as well as loss of descending inhibition through noradrenergic and serotonergic fibers located in the dorsolateral funiculus (Watkins et al., 1984; Faden et al., 1988; Liu et al., 1990). This increased excitability can be reduced by administration of the GABA_B_ agonist baclofen (Hao et al., 1992) and serotonergic/noradrenergic drugs (Barbeau and Norman, 2003; Hains et al., 2003).

Interestingly, the expression of spinal LTP is normally inhibited by descending pathways (Sandkuhler and Liu, 1998; Gjerstad et al., 2001). Using our spinal training paradigm, we showed that intermittent stimulation given *prior* to complete transection was not sufficient to produce a learning deficit, indicating a brain-mediated descending protection against the deleterious effects of intermittent stimulation. Further, we showed the necessity for the DLF in this effect. We lesioned the DLF at T2, then gave intermittent stimulation followed by a complete transection at T8, and found that by removing the protection of this descending system, rats exhibited a spinal learning deficit (Crown and Grau, 2005). Similarly, as SCI may disrupt these fibers, it is possible that the vulnerability to nociceptive plasticity after injury reflects a loss of descending inhibition (Hains et al., 2002). Thus, spinal injury may create a predisposition toward the lasting maladaptive effects of nociceptive input. This notion has even greater clinical impact, when one considers the potential blockade of descending inhibition conferred by surgical anesthesia. To test this, Washburn et al. (2007) assessed the effect of the anesthetic pentobarbital on the stimulus-induced spinal learning deficit. We first delivered 6 min of intermittent uncontrollable stimulation (AC 60 Hz, 1.5 mA) to rats that were under pentobarbital anesthesia, 24 h after complete T2 transection. The next day these rats were tested for spinal instrumental learning. These rats failed to learn, indicating that pentobarbital anesthesia does not protect against the spinal mechanisms responsible for the induction of the stimulation-induced learning deficit. We next assessed whether pentobarbital anesthesia could protect against the induction of the learning deficit if *intact* rats were given intermittent uncontrollable stimulation under anesthesia. Intact rats were given an injection of either pentobarbital or saline vehicle, followed by 6 min of uncontrollable intermittent stimulation to the tail and the next day all rats were given a complete transection, followed 24 h later with spinal instrumental testing. Rats that were not under anesthesia during the intermittent uncontrollable stimulation (saline-treated), did not exhibit a learning deficit, replicating the earlier finding from Crown and Grau (2005) that descending supraspinal input provides protection against the stimulation-induced deficit. Interestingly, those rats that were under pentobarbital anesthesia during the intermittent uncontrollable stimulation did exhibit a learning deficit when later tested, indicating that the descending protection was undermined by anesthesia, leaving the spinal cord susceptible to the induction of maladaptive nociceptive plasticity. These findings highlight how important it is to be cognizant of the capacity of the spinal cord for nociceptive plasticity, particularly when supraspinal controls are removed under anesthesia. The pain memory that can be formed during surgical procedures could contribute to the development of neuropathic pain, and in the instance of SCI, could undermine functional recovery.

From our studies detailing the varying stimulation parameters that produce divergent effects on adaptive and maladaptive forms of spinal plasticity, it is clear that the timing and controllability of stimulation are very important predictors of recovery of function. Harnessing the specific capacity for spinal cord learning may improve rehabilitative training of limb position after SCI. The neurobiological basis of training-induced plasticity within the spinal cord has been studied in some detail. For example, following chronic complete SCI there is an increase in glycinergic (de Leon et al., 1999; Cantoria et al., 2012) and GABAergic inhibitory tone (Edgerton et al., 2001; Tillakaratne et al., 2002) that has been shown to hinder locomotor performance. Systemic administration of the glycinergic antagonist strychnine improves locomotion in untrained animals, whereas animals with previous step training do not require strychnine (de Leon et al., 1999). Moreover, strychnine does not yield further improvement beyond step-training alone, suggesting that spinal training has the capacity to modulate inhibitory neurotransmitter levels in the spinal cord. However, the type of training used has a profound influence on glycinergic tone. If animals are re-trained to stand after treadmill step training, there is a reemergence of glycinergic inhibition, resulting in poor treadmill performance that was reversible by strychnine.

Indeed the locomotor physiology literature has produced substantial evidence that following complete transection the spinal cord can be trained to elicit stepping (for review see Edgerton et al., 1997). For example, Lovely et al. (1990) found that treadmill training can improve stepping in spinally transected cats. A similar improvement can be quantified through the use of kinematics and electromyography (EMG) in rats and mice, and consistent differences have been observed between untrained and trained animals in a variety of different contexts (Drew and Rossignol, 1987; Roy et al., 1998; Courtine et al., 2009; van den Brand et al., 2012). Importantly, motor training after SCI has been shown to be exquisitely task-specific; training on one task can interfere with training on other tasks (Garcia-Alias et al., 2009; de Leon et al., 2011). Collaborative work has revealed that rats that are trained to stand on a treadmill have impaired instrumental learning (Bigbee et al., 2007). Interestingly, stand training is also known to interfere with locomotor training after SCI (de Leon et al., 1999), suggesting that instrumental training in the Horridge paradigm may be able to predict locomotor recovery. These findings illustrate how critical it is to control the types of input that are received after SCI. A study from Petruska et al. (2007) highlighted this importance. They trained transected rats to walk on a treadmill, and found that this training regimen successfully improved locomotor performance in most of the trained animals. Interestingly, a subset of rats was found to have lesions on their hindpaws and the beneficial effects of training were markedly diminished in these rats. This finding suggests that the noxious input that these animals were receiving may have been undermining their capacity for adaptive locomotor plasticity. Further, recent research indicates that even manipulations that are considered relatively passive or innocuous may be inducing unintended maladaptive plasticity, and doing untold damage to recovery of function. Studies in the human patient population of the efficacy of relatively passive rehabilitative efforts after SCI (including stretching to increase range of motion) have revealed that these interventions may have few beneficial effects on locomotor recovery (Harvey et al., 2002, 2009). It has been shown in rodents that peripheral input such as leg immobilization in a wheelchair model, muscle stretching, or repeated tactile stimulation produce lasting changes in spinal circuitry that can impair recovery (Hutchinson et al., 2004; Hoschouer et al., 2010; Caudle et al., 2011).

## Summary

**Figure 8:**
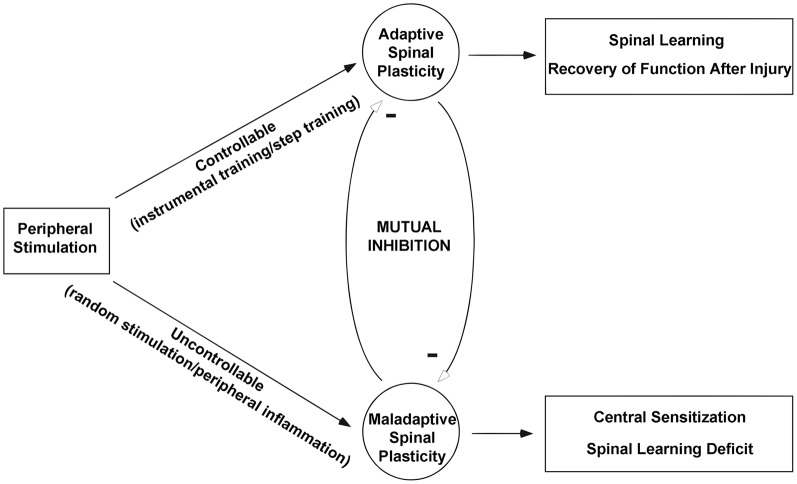
**Theoretical framework for understanding the relationship between adaptive and maladaptive spinal cord plasticity.** After spinal cord injury peripheral stimulation can take a controllable form such as instrumental training or step training that promotes adaptive plasticity and leads to further improvements in spinal learning and recovery of function. On the other hand peripheral stimulation delivered in an uncontrollable form such as random (position-independent) stimulation or peripheral inflammation produces a maladaptive spinal plasticity that promotes central sensitization and deficits in future spinal cord learning. Modified from Ferguson et al. (2006).

The existing literature indicates many similarities between central sensitization within spinal pain pathways and stimulus-induced deficits in spinal learning after SCI. Taken together the findings indicate that central nociceptive sensitization and adaptive spinal learning are opposing forms of spinal plasticity (Figure 8). Adaptive training of the spinal cord undermines the development of central sensitization, and conversely central nociceptive stimulation undermines the capacity for spinal learning.

While studies of spinal cord plasticity after SCI have uncovered a remarkable degree of plasticity, it must also be remembered that this plasticity is a two-edged sword; adaptive processes can foster recovery and reduce neuropathic pain while maladaptive mechanisms have the opposite effect. This review has aimed to bring to light the ongoing struggle between nociceptive and adaptive forms of spinal plasticity after injury. We have shown that certain peripheral stimuli limit adaptive spinal learning and may actually promote maladaptive forms of spinal plasticity. This maladaptive plasticity may include hyperexcitability of spinal nociceptive systems, leading to pain and spasticity. Maladaptive spinal plasticity may ultimately lead to intractable pain in SCI, a common problem for SCI patients (for review see Yezierski, 1996, 2000, 2009; Bruce et al., 2002; Weaver et al., 2002; Finnerup and Jensen, 2004; Crown and Grau, 2005; Crown et al., 2006, 2008; Finnerup et al., 2007).

The present paper has focused on the negative side of spinal plasticity: the mechanisms by which inappropriate spinal training (uncontrollable electrical stimulation) and persistent nociceptive stimuli can impair spinal cord learning. In the companion paper in this issue Grau et al. (2012) discuss the positive side to spinal training: the mechanisms by which specific types of spinal cord training can protect against the development of nociceptive plasticity and may promote spinal adaptation and recovery of function after SCI. An effective rehabilitation strategy will integrate the messages from both papers to maximize positive elements of spinal cord training while limiting the negative consequences of inappropriate stimulus timing and nociceptive input below the injury. Ongoing work is focused on determining the specific conditions that limit maladaptive plasticity, while promoting adaptive spinal plasticity. This work will be critical in order to tailor rehabilitative and restorative therapies toward maximizing recovery of function after SCI.

### Conflict of interest statement

Dr. Crown is currently employed by Abbott Laboratories. The current research is not affiliated with his current duties at Abbott Laboratories. The other authors declare that the research was conducted in the absence of any commercial or financial relationships that could be construed as a potential conflict of interest.

## References

[B1] Abraham W. C. Bear M. F. (1996). Metaplasticity: the plasticity of synaptic plasticity. Trends Neurosci. 19, 126–130 10.1007/978-3-540-88955-7_6 8658594

[B2] Akers R. F. Lovinger D. M. Colley P. A. Linden D. J. Routtenberg A. (1986). Translocation of protein kinase C activity may mediate hippocampal long-term potentiation. Science 231, 587–589 10.1126/science.3003904 3003904

[B3] Allen N. J. Barres B. A. (2005). Signaling between glia and neurons: focus on synaptic plasticity. Curr. Opin. Neurobiol. 15, 542–548 10.1016/j.conb.2005.08.006 16144764

[B4] Andersen O. K. Finnerup N. B. Spaich E. G. Jensen T. S. Arendt-Nielsen L. (2004). Expansion of nociceptive withdrawal reflex receptive fields in spinal cord injured humans. Clin. Neurophysiol. 115, 2798–2810 10.1016/j.clinph.2004.07.003 15546788

[B5] Barbeau H. Norman K. E. (2003). The effect of noradrenergic drugs on the recovery of walking after spinal cord injury. Spinal Cord 41, 137–143 10.1038/sj.sc.3101374 12612615

[B6] Barbeau H. Rossignol S. (1987). Recovery of locomotion after chronic spinalization in the adult cat. Brain Res. 412, 84–95 10.1016/0006-8993(87)91442-9 3607464

[B7] Basso D. M. Beattie M. S. Bresnahan J. C. (1995). A sensitive and reliable locomotor rating scale for open field testing in rats. J. Neurotrauma 12, 1–21 7783230 10.1089/neu.1995.12.1

[B8] Baumbauer K. M. Hoy K. C. Jr. Huie J. R. Hughes A. J. Woller S. A. Puga D. A. (2008). Timing in the absence of supraspinal input I: variable, but not fixed, spaced stimulation of the sciatic nerve undermines spinally-mediated instrumental learning. Neuroscience 155, 1030–1047 10.1016/j.neuroscience.2008.07.003 18674601 PMC2633135

[B9] Baumbauer K. M. Huie J. R. Hughes A. J. Grau J. W. (2009). Timing in the absence of supraspinal input II: regularly spaced stimulation induces a lasting alteration in spinal function that depends on the NMDA receptor, BDNF release, and protein synthesis. J. Neurosci. 29, 14383–14393 10.1523/JNEUROSCI.3583-09.2009 19923273 PMC2800823

[B10] Baumbauer K. M. Young E. E. Hoy K. C. Jr. France J. L. Joynes R. L. (2006). Intrathecal infusions of anisomycin impact the learning deficit but not the learning effect observed in spinal rats that have received instrumental training. Behav. Brain Res. 173, 299–309 10.1016/j.bbr.2006.06.041 16914213

[B11] Baumbauer K. M. Young E. E. Hoy K. C. Jr. Joynes R. L. (2007). Neurokinin receptors modulate the impact of uncontrollable stimulation on adaptive spinal plasticity. Behav. Neurosci. 121, 1082–1094 10.1037/0735-7044.121.5.1082 17907839

[B12] Beattie E. C. Stellwagen D. Morishita W. Bresnahan J. C. Ha B. K. Von Zastrow M. (2002). Control of synaptic strength by glial TNFalpha. Science 295, 2282–2285 10.1126/science.1067859 11910117

[B13] Bennett A. D. Chastain K. M. Hulsebosch C. E. (2000). Alleviation of mechanical and thermal allodynia by CGRP(8-37) in a rodent model of chronic central pain. Pain 86, 163–175 10779673 10.1016/s0304-3959(00)00242-6

[B14] Beric A. (1993). Central pain: “new” syndromes and their evaluation. Muscle Nerve 16, 1017–1024 10.1002/mus.880161004 8413354

[B15] Beric A. Dimitrijevic M. R. Lindblom U. (1988). Central dysesthesia syndrome in spinal cord injury patients. Pain 34, 109–116 3174149 10.1016/0304-3959(88)90155-8

[B16] Bigbee A. J. Crown E. D. Ferguson A. R. Roy R. R. Tillakaratne N. J. Grau J. W. (2007). Two chronic motor training paradigms differentially influence acute instrumental learning in spinally transected rats. Behav. Brain Res. 180, 95–101 10.1016/j.bbr.2007.02.029 17434606 PMC2234650

[B17] Bliss T. V. Lomo T. (1973). Long-lasting potentiation of synaptic transmission in the dentate area of the anaesthetized rabbit following stimulation of the perforant path. J. Physiol. 232, 331–356 4727084 10.1113/jphysiol.1973.sp010273 PMC1350458

[B18] Boyce V. S. Park J. Gage F. H. Mendell L. M. (2012). Differential effects of brain-derived neurotrophic factor and neurotrophin-3 on hindlimb function in paraplegic rats. Eur. J. Neurosci. 35, 221–232 10.1111/j.1460-9568.2011.07950.x 22211901 PMC3509221

[B19] Bruce J. C. Oatway M. A. Weaver L. C. (2002). Chronic pain after clip-compression injury of the rat spinal cord. Exp. Neurol. 178, 33–48 10.1006/exnr.2002.8026 12460606

[B20] Buerger A. A. Fennessy A. (1970). Learning of leg position in chronic spinal rats. Nature 225, 751–752 5412784 10.1038/225751a0

[B21] Cai L. Courtine G. Fong A. Burdick J. Roy R. Edgerton V. (2006). Plasticity of functional connectivity in the adult spinal cord. Philos. Trans. R. Soc. Lond. B Biol. Sci. 361, 1635–1646 10.1098/rstb.2006.1884 16939979 PMC1664672

[B22] Cantoria M. J. See P. A. Singh H. De Leon R. D. (2012). Adaptations in glutamate and glycine content within the lumbar spinal cord are associated with the generation of novel gait patterns in rats following neonatal spinal cord transection. J. Neurosci. 31, 18598–18605 10.1523/JNEUROSCI.3499-11.2011 22171058 PMC3268368

[B23] Carew T. J. Walters E. T. Kandel E. R. (1981). Associative learning in Aplysia: cellular correlates supporting a conditioned fear hypothesis. Science 211, 501–504 10.1126/science.7455692 7455692

[B24] Carlton S. M. Du J. Tan H. Y. Nesic O. Hargett G. L. Bopp A. C. (2009). Peripheral and central sensitization in remote spinal cord regions contribute to central neuropathic pain after spinal cord injury. Pain 147, 265–276 10.1016/j.pain.2009.09.030 19853381 PMC2787843

[B25] Caudle K. L. Brown E. H. Shum-Siu A. Burke D. A. Magnuson T. S. Voor M. J. (2011). Hindlimb immobilization in a wheelchair alters functional recovery following contusive spinal cord injury in the adult rat. Neurorehabil. Neural Repair 25, 729–739 10.1177/1545968311407519 21697451 PMC4419333

[B26] Choi J. I. Svensson C. I. Koehrn F. J. Bhuskute A. Sorkin L. S. (2010). Peripheral inflammation induces tumor necrosis factor dependent AMPA receptor trafficking and Akt phosphorylation in spinal cord in addition to pain behavior. Pain 149, 243–253 10.1016/j.pain.2010.02.008 20202754 PMC2860679

[B27] Coderre T. J. Melzack R. (1992). The role of NMDA receptor-operated calcium channels in persistent nociception after formalin-induced tissue injury. J. Neurosci. 12, 3671–3675 1326611 10.1523/JNEUROSCI.12-09-03671.1992 PMC6575721

[B28] Collingridge G. L. Bliss T. V. P. (1987). NMDA receptors - their role in long-term potentiation. Trends Neurosci. 10, 288–293

[B29] Courtine G. Gerasimenko Y. van den Brand R. Yew A. Musienko P. Zhong H. (2009). Transformation of nonfunctional spinal circuits into functional states after the loss of brain input. Nat. Neurosci. 12, 1333–1342 10.1038/nn.2401 19767747 PMC2828944

[B30] Crown E. D. Ferguson A. R. Joynes R. L. Grau J. W. (2002a). Instrumental learning within the spinal cord: IV. Induction and retention of the behavioral deficit observed after noncontingent shock. Behav. Neurosci. 116, 1032–1051 12492302 10.1037//0735-7044.116.6.1032

[B31] Crown E. D. Ferguson A. R. Joynes R. L. Grau J. W. (2002b). Instrumental learning within the spinal cord. II. Evidence for central mediation. Physiol. Behav. 77, 259–267 10.1016/S0031-9384(02)00859-4 12419402

[B32] Crown E. D. Grau J. W. (2001). Preserving and restoring behavioral potential within the spinal cord using an instrumental training paradigm. J. Neurophysiol. 86, 845–855 11495955 10.1152/jn.2001.86.2.845

[B33] Crown E. D. Grau J. W. (2005). Evidence that descending serotonergic systems protect spinal cord plasticity against the disruptive effect of uncontrollable stimulation. Exp. Neurol. 196, 164–176 10.1016/j.expneurol.2005.07.016 16139268

[B34] Crown E. D. Gwak Y. S. Ye Z. Johnson K. M. Hulsebosch C. E. (2008). Activation of p38 MAP kinase is involved in central neuropathic pain following spinal cord injury. Exp. Neurol. 213, 257–267 10.1016/j.expneurol.2008.05.025 18590729 PMC2580737

[B35] Crown E. D. Ye Z. Johnson K. M. Xu G. Y. McAdoo D. J. Hulsebosch C. E. (2006). Increases in the activated forms of ERK 1/2, p38 MAPK, and CREB are correlated with the expression of at-level mechanical allodynia following spinal cord injury. Exp. Neurol. 199, 397–407 10.1016/j.expneurol.2006.01.003 16478624

[B36] Davidoff G. Roth E. Guarracini M. Sliwa J. Yarkony G. (1987). Function-limiting dysesthetic pain syndrome among traumatic spinal cord injury patients: a cross-sectional study. Pain 29, 39–48 10.1016/0304-3959(87)90176-X 3588000

[B37] de Leon R. D. Hodgson J. A. Roy R. R. Edgerton V. R. (1998). Locomotor capacity attributable to step training versus spontaneous recovery after spinalization in adult cats. J. Neurophysiol. 79, 1329–1340 9497414 10.1152/jn.1998.79.3.1329

[B38] de Leon R. D. See P. A. Chow C. H. (2011). Differential effects of low versus high amounts of weight supported treadmill training in spinally transected rats. J. Neurotrauma 28, 1021–1033 10.1089/neu.2010.1699 21476782 PMC3113444

[B39] de Leon R. D. Tamaki H. Hodgson J. A. Roy R. R. Edgerton V. R. (1999). Hindlimb locomotor and postural training modulates glycinergic inhibition in the spinal cord of the adult spinal cat. J. Neurophysiol. 82, 359–369 10400964 10.1152/jn.1999.82.1.359

[B40] Derkach V. Barria A. Soderling T. R. (1999). Ca2+/calmodulin-kinase II enhances channel conductance of alpha-amino-3-hydroxy-5-methyl-4-isoxazolepropionate type glutamate receptors. Proc. Natl. Acad. Sci. U.S.A. 96, 3269 10.1073/pnas.96.6.3269 10077673 PMC15931

[B41] Dietz V. Harkema S. J. (2004). Locomotor activity in spinal cord-injured persons. J. Appl. Physiol. 96, 1954–1960 10.1152/japplphysiol.00942.2003 15075315

[B42] Dougherty P. M. Palecek J. Paleckova V. Sorkin L. S. Willis W. D. (1992). The role of NMDA and non-NMDA excitatory amino acid receptors in the excitation of primate spinothalamic tract neurons by mechanical, chemical, thermal, and electrical stimuli. J. Neurosci. 12, 3025–3041 1353793 10.1523/JNEUROSCI.12-08-03025.1992 PMC6575659

[B43] Drdla-Schutting R. Benrath J. Wunderbaldinger G. Sandkuhler J. (2012). Erasure of a spinal memory trace of pain by a brief, high-dose opioid administration. Science 335, 235–238 10.1126/science.1211726 22246779

[B44] Drew T. Rossignol S. (1987). A kinematic and electromyographic study of cutaneous reflexes evoked from the forelimb of unrestrained walking cats. J. Neurophysiol. 57, 1160–1184 3585458 10.1152/jn.1987.57.4.1160

[B45] Edgerton V. R. Courtine G. Gerasimenko Y. P. Lavrov I. Ichiyama R. M. Fong A. J. (2008). Training locomotor networks. Brain Res. Rev. 57, 241–254 10.1016/j.brainresrev.2007.09.002 18022244 PMC2288528

[B46] Edgerton V. R. de Leon R. D. Tillakaratne N. Recktenwald M. R. Hodgson J. A. Roy R. R. (1997). Use-dependent plasticity in spinal stepping and standing. Adv. Neurol. 72, 233–247 8993702

[B47] Edgerton V. R. Leon R. D. Harkema S. J. Hodgson J. A. London N. Reinkensmeyer D. J. (2001). Retraining the injured spinal cord. J. Physiol. 533, 15–22 10.1111/j.1469-7793.2001.0015b.x 11351008 PMC2278598

[B48] Edgerton V. R. Tillakaratne N. J. K. Bigbee A. J. de Leon R. D. Roy R. R. (2004). Plasticity of the spinal neural circuitry after injury. Annu. Rev. Neurosci. 27, 145–167 10.1146/annurev.neuro.27.070203.144308 15217329

[B49] Faden A. I. Gannon A. Basbaum A. I. (1988). Use of serotonin immunocytochemistry as a marker of injury severity after experimental spinal trauma in rats. Brain Res. 450, 94–100 10.1016/0006-8993(88)91548-X 3401725

[B50] Ferguson A. R. Bolding K. A. Huie J. R. Hook M. A. Santillano D. R. Miranda R. C. (2008a). Group I metabotropic glutamate receptors control metaplasticity of spinal cord learning through a protein kinase C-dependent mechanism. J. Neurosci. 28, 11939–11949 10.1523/JNEUROSCI.3098-08.2008 19005059 PMC2628285

[B51] Ferguson A. R. Christensen R. N. Gensel J. C. Miller B. A. Sun F. Beattie E. C. (2008b). Cell death after spinal cord injury is exacerbated by rapid TNF alpha-induced trafficking of GluR2-lacking AMPARs to the plasma membrane. J. Neurosci. 28, 11391–11400 10.1523/JNEUROSCI.3708-08.2008 18971481 PMC2598739

[B52] Ferguson A. R. Crown E. D. Grau J. W. (2006). Nociceptive plasticity inhibits adaptive learning in the spinal cord. Neuroscience 141, 421–431 10.1016/j.neuroscience.2006.03.029 16678969

[B53] Ferguson A. R. Hook M. A. Garcia G. Bresnahan J. C. Beattie M. S. Grau J. W. (2004). A simple *post hoc* transformation that improves the metric properties of the BBB scale for rats with moderate to severe spinal cord injury. J. Neurotrauma 21, 1601–1613 10.1089/neu.2004.21.1601 15684652

[B50a] Ferguson A. R. Huie J. R. Crown E. D. Grau J. W. (2012). Central nociceptive sensitization vs. spinal cord training: opposing forms of plasticity that dictate function after complete spinal cord injury. Front. Physiol. 3:396 10.3389/fphys.2012.00396 PMC3463829 23060820

[B54] Finnerup N. B. Jensen T. S. (2004). Spinal cord injury pain–mechanisms and treatment. Eur. J. Neurol. 11, 73–82 10.1046/j.1351-5101.2003.00725.x 14748766

[B55] Finnerup N. B. Johannesen I. L. Bach F. W. Jensen T. S. (2003). Sensory function above lesion level in spinal cord injury patients with and without pain. Somatosens. Mot. Res. 20, 71–76 10.1080/0899022031000083843 12745445

[B56] Finnerup N. B. Sorensen L. Biering-Sorensen F. Johannesen I. L. Jensen T. S. (2007). Segmental hypersensitivity and spinothalamic function in spinal cord injury pain. Exp. Neurol. 207, 139–149 10.1016/j.expneurol.2007.06.001 17628539

[B57] Fisher K. Coderre T. J. (1996a). Comparison of nociceptive effects produced by intrathecal administration of mGluR agonists. Neuroreport 7, 2743–2747 8981459 10.1097/00001756-199611040-00067

[B58] Fisher K. Coderre T. J. (1996b). The contribution of metabotropic glutamate receptors (mGluRs) to formalin-induced nociception. Pain 68, 255–263 9121812 10.1016/s0304-3959(96)03212-5

[B59] Fisher K. Lefebvre C. Coderre T. J. (2002). Antinociceptive effects following intrathecal pretreatment with selective metabotropic glutamate receptor compounds in a rat model of neuropathic pain. Pharmacol. Biochem. Behav. 73, 411–418 10.1016/S0091-3057(02)00832-8 12117596

[B60] Fitzgerald L. A. Thompson R. F. (1967). Classical conditioning of the hindlimb flexion reflex in the acute spinal cat. Psychon. Sci. 47, 345–351

[B61] Fritsch B. Reis J. Martinowich K. Schambra H. M. Ji Y. Cohen L. G. (2010). Direct current stimulation promotes BDNF-dependent synaptic plasticity: potential implications for motor learning. Neuron 66, 198–204 10.1016/j.neuron.2010.03.035 20434997 PMC2864780

[B62] Fundytus M. E. Osborne M. G. Henry J. L. Coderre T. J. Dray A. (2002). Antisense oligonucleotide knockdown of mGluR1 alleviates hyperalgesia and allodynia associated with chronic inflammation. Pharmacol. Biochem. Behav. 73, 401–410 10.1016/S0091-3057(02)00831-6 12117595

[B63] Furukawa K. Mattson M. P. (1998). The transcription factor NF-kappaB mediates increases in calcium currents and decreases in NMDA- and AMPA/kainate-induced currents induced by tumor necrosis factor-alpha in hippocampal neurons. J. Neurochem. 70, 1876–1886 10.1046/j.1471-4159.1998.70051876.x 9572271

[B64] Galan A. Laird J. Cervero F. (2004). *In vivo* recruitment by painful stimuli of AMPA receptor subunits to the plasma membrane of spinal cord neurons. Pain 112, 315–323 10.1016/j.pain.2004.09.011 15561387

[B65] Garcia-Alias G. Barkhuysen S. Buckle M. Fawcett J. W. (2009). Chondroitinase ABC treatment opens a window of opportunity for task-specific rehabilitation. Nat. Neurosci. 12, 1145–1151 10.1038/nn.2377 19668200

[B65a] Garraway S. M. Woller S. A. Huie J. R. Hook M. A. Huang Y. J. Hartman J. (2012). Peripheral Noxious Stimulation Following Contusion Spinal Cord Injury Increases the Incidence of Mechanical Allodynia and the Expression of TNF Alpha in the Spinal Cord. 2012 Neuroscience Meeting Planner. New Orleans, LA: Society for Neuroscience

[B66] Gjerstad J. Tjolsen A. Hole K. (2001). Induction of long-term potentiation of single wide dynamic range neurones in the dorsal horn is inhibited by descending pathways. Pain 91, 263–268 11275383 10.1016/S0304-3959(00)00448-6

[B67] Gomez-Pinilla F. Huie J. Ying Z. Ferguson A. Crown E. Baumbauer K. (2007). BDNF and learning: evidence that instrumental training promotes learning within the spinal cord by up-regulating BDNF expression. Neuroscience 148, 893–906 10.1016/j.neuroscience.2007.05.051 17719180 PMC3225191

[B68] Gorio A. Madaschi L. Di Stefano B. Carelli S. Di Giulio A. M. De Biasi S. (2005). Methylprednisolone neutralizes the beneficial effects of erythropoietin in experimental spinal cord injury. Proc. Natl. Acad. Sci. U.S.A. 102, 16379–16384 10.1016/j.neuroscience.2006.10.023 16260722 PMC1283477

[B69] Grau J. W. Barstow D. G. Joynes R. L. (1998). Instrumental learning within the spinal cord: I. Behavioral properties. Behav. Neurosci. 112, 1366–1386 9926819 10.1037//0735-7044.112.6.1366

[B70] Grau J. W. Crown E. D. Ferguson A. R. Washburn S. N. Hook M. A. Miranda R. C. (2006). Instrumental learning within the spinal cord: underlying mechanisms and implications for recovery after injury. Behav. Cogn. Neurosci. Rev. 5, 191–239 10.1177/1534582306289738 17099112

[B71] Grau J. W. Huie R. Garraway S. M. Hook M. A. Crown E. D. Baumbauer K. M. (2012). Impact of behavioral control on the processing of nociceptive stimulation. Front. Physiol. 3:262 10.3389/fphys.2012.00262 22934018 PMC3429038

[B72] Grau J. W. Patterson M. M. (eds.). (2001). Spinal Cord Plasticity: Alterations in Reflex Function. Boston, MA: Kluwer Academic

[B73] Grau J. W. Washburn S. N. Hook M. A. Ferguson A. R. Crown E. D. Garcia G. (2004). Uncontrollable stimulation undermines recovery after spinal cord injury. J. Neurotrauma 21, 1795–1817 10.1089/neu.2004.21.1795 15684770

[B73a] Grillner S. (1975). Locomotion in vertebrates: central mechanisms and reflex interaction. Physiol. Rev. 55, 247–304 1144530 10.1152/physrev.1975.55.2.247

[B74] Grillner S. Zangger P. (1979). On the central generation of locomotion in the low spinal cat. Exp. Brain. Res. 34, 241–261 421750 10.1007/BF00235671

[B75] Groves P. M. De Marco R. Thompson R. F. (1969). Habituation and sensitization of spinal interneuron activity in acute spinal cat. Brain Res. 14, 521–525 5794923 10.1016/0006-8993(69)90129-2

[B76] Groves P. M. Thompson R. F. (1970). Habituation: a dual-process theory. Psychol. Rev. 77, 419 4319167 10.1037/h0029810

[B77] Gruner J. A. (1992). A monitored contusion model of spinal cord injury in the rat. J. Neurotrauma 9, 123–126 discussion: 126–128. 1404425 10.1089/neu.1992.9.123

[B78] Gwak Y. S. Unabia G. C. Hulsebosch C. E. (2009). Activation of p-38alpha MAPK contributes to neuronal hyperexcitability in caudal regions remote from spinal cord injury. Exp. Neurol. 220, 154–161 10.1016/j.expneurol.2009.08.012 19699199 PMC3008350

[B79] Hains B. C. Everhart A. W. Fullwood S. D. Hulsebosch C. E. (2002). Changes in serotonin, serotonin transporter expression and serotonin denervation supersensitivity: involvement in chronic central pain after spinal hemisection in the rat. Exp. Neurol. 175, 347–362 10.1006/exnr.2002.7892 12061865

[B80] Hains B. C. Willis W. D. Hulsebosch C. E. (2003). Serotonin receptors 5-HT1A and 5-HT3 reduce hyperexcitability of dorsal horn neurons after chronic spinal cord hemisection injury in rat. Exp. Brain Res. 149, 174–186 10.1007/s00221-002-1352-x 12610685

[B81] Hains B. C. Yucra J. A. Hulsebosch C. E. (2001). Reduction of pathological and behavioral deficits following spinal cord contusion injury with the selective cyclooxygenase-2 inhibitor NS-398. J. Neurotrauma 18, 409–423 10.1089/089771501750170994 11336442

[B82] Hao J. X. Xu X. J. Yu Y. X. Seiger A. Wiesenfeld-Hallin Z. (1992). Baclofen reverses the hypersensitivity of dorsal horn wide dynamic range neurons to mechanical stimulation after transient spinal cord ischemia; implications for a tonic GABAergic inhibitory control of myelinated fiber input. J. Neurophysiol. 68, 392–396 1527566 10.1152/jn.1992.68.2.392

[B83] Hargreaves K. Dubner R. Brown F. Flores C. Joris J. (1988). A new and sensitive method for measuring thermal nociception in cutaneous hyperalgesia. Pain 32, 77–88 10.1016/0304-3959(88)90026-7 3340425

[B84] Harkema S. Gerasimenko Y. Hodes J. Burdick J. Angeli C. Chen Y. (2011). Effect of epidural stimulation of the lumbosacral spinal cord on voluntary movement, standing, and assisted stepping after motor complete paraplegia: a case study. Lancet 377, 1938–1947 10.1016/S0140-6736(11)60547-3 21601270 PMC3154251

[B85] Harvey L. Herbert R. Crosbie J. (2002). Does stretching induce lasting increases in joint ROM? A systematic review. Physiother. Res. Int. 7, 1–13 11992980 10.1002/pri.236

[B86] Harvey L. A. Lin C. W. Glinsky J. V. De Wolf A. (2009). The effectiveness of physical interventions for people with spinal cord injuries: a systematic review. Spinal Cord 47, 184–195 10.1038/sc.2008.100 18725889

[B87] Hermann G. E. Rogers R. C. Bresnahan J. C. Beattie M. S. (2001). Tumor necrosis factor-alpha induces cFOS and strongly potentiates glutamate-mediated cell death in the rat spinal cord. Neurobiol. Dis. 8, 590–599 10.1006/nbdi.2001.0414 11493024

[B88] Hook M. A. Huie J. R. Grau J. W. (2008). Peripheral inflammation undermines the plasticity of the isolated spinal cord. Behav. Neurosci. 122, 233–249 10.1037/0735-7044.122.1.233 18298266 PMC2665167

[B89] Horridge G. A. (1962). Learning of leg position by headless insects. Nature 193, 697–698 14449018 10.1038/193697a0

[B90] Hoschouer E. L. Finseth T. Flinn S. Basso D. M. Jakeman L. B. (2010). Sensory stimulation prior to spinal cord injury induces post-injury dysesthesia in mice. J. Neurotrauma 27, 777–787 10.1089/neu.2009.1182 20121420 PMC2943942

[B91] Hoy K. C. Huie J. R. Grau J. W. (2012). AMPA receptor mediated behavioral plasticity in the isolated rat spinal cord. Brain Behav. Res. [Epub ahead of print]. 10.1016/j.bbr.2012.09.007 22982187 PMC3482296

[B92] Hubscher C. H. Johnson R. D. (2000). Effects of acute and chronic midthoracic spinal cord injury on neural circuits for male sexual function. II. Descending pathways. J. Neurophysiol. 83, 2508–2518 10805652 10.1152/jn.2000.83.5.2508

[B93] Huie J. R. Baumbauer K. M. Lee K. H. Bresnahan J. C. Beattie M. S. Ferguson A. R. (2012a). Glial tumor necrosis factor alpha (TNFalpha) generates metaplastic inhibition of spinal learning. PLoS ONE 7:e39751 10.1371/journal.pone.0039751 22745823 PMC3379985

[B94] Huie J. R. Garraway S. M. Baumbauer K. M. Hoy K. C. Jr. Beas B. S. Montgomery K. S. (2012b). Brain-derived neurotrophic factor promotes adaptive plasticity within the spinal cord and mediates the beneficial effects of controllable stimulation. Neuroscience 200C, 74–90 10.1016/j.neuroscience.2011.10.028 22056599 PMC3249495

[B95] Hulsebosch C. E. Hains B. C. Crown E. D. Carlton S. M. (2009). Mechanisms of chronic central neuropathic pain after spinal cord injury. Brain Res. Rev. 60, 202–213 10.1016/j.brainresrev.2008.12.010 19154757 PMC2796975

[B96] Hutchinson K. J. Gomez-Pinilla F. Crowe M. J. Ying Z. Basso D. M. (2004). Three exercise paradigms differentially improve sensory recovery after spinal cord contusion in rats. Brain 127, 1403–1414 10.1093/brain/awh160 15069022

[B97] Ji R. R. Kohno T. Moore K. A. Woolf C. J. (2003). Central sensitization and LTP: do pain and memory share similar mechanisms? Trends Neurosci. 26, 696–705 10.1016/j.tins.2003.09.017 14624855

[B98] Jia D. Gao G. D. Liu Y. He S. M. Zhang X. N. Zhang Y. F. (2007). TNFa involves in altered prefrontal synaptic transmission in mice with persistent inflammatory pain. Neurosci. Lett. 415, 1–5 10.1016/j.neulet.2006.12.032 17222972

[B99] Jindrich D. L. Joseph M. S. Otoshi C. K. Wei R. Y. Zhong H. Roy R. R. (2009). Spinal learning in the adult mouse using the Horridge paradigm. J. Neurosci. Methods 182, 250–254 10.1016/j.jneumeth.2009.06.001 19520117 PMC2727573

[B100] Joynes R. L. Ferguson A. R. Crown E. D. Patton B. C. Grau J. W. (2003). Instrumental learning within the spinal cord: V. Evidence the behavioral deficit observed after noncontingent nociceptive stimulation reflects an intraspinal modification. Behav. Brain Res. 141, 159–170 10.1016/S0166-4328(02)00372-8 12742252

[B101] Joynes R. L. Janjua K. Grau J. W. (2004). Instrumental learning within the spinal cord: VI. The NMDA receptor antagonist, AP5, disrupts the acquisition and maintenance of an acquired flexion response. Behav. Brain Res. 154, 431–438 10.1016/j.bbr.2004.03.030 15313031

[B102] Lee H. K. Barbarosie M. Kameyama K. Bear M. F. Huganir R. L. (2000). Regulation of distinct AMPA receptor phosphorylation sites during bidirectional synaptic plasticity. Nature 405, 955–958 10.1038/35016089 10879537

[B103] Leem J. W. Kim H. K. Hulsebosch C. E. Gwak Y. S. (2010). Ionotropic glutamate receptors contribute to maintained neuronal hyperexcitability following spinal cord injury in rats. Exp. Neurol. 224, 321–324 10.1016/j.expneurol.2010.02.012 20211179 PMC3008557

[B104] Leonoudakis D. Braithwaite S. P. Beattie M. S. Beattie E. C. (2004). TNFa-induced AMPA-receptor trafficking in CNS neurons; relevance to excitotoxicity? Neuron Glia Biol. 1, 263 16520832 10.1017/S1740925X05000608 PMC1389713

[B105] Leonoudakis D. Zhao P. Beattie E. C. (2008). Rapid tumor necrosis factor alpha-induced exocytosis of glutamate receptor 2-lacking AMPA receptors to extrasynaptic plasma membrane potentiates excitotoxicity. J. Neurosci. 28, 2119–2130 10.1523/JNEUROSCI.5159-07.2008 18305246 PMC6671833

[B106] Liu D. X. Valadez V. Sorkin L. S. McAdoo D. J. (1990). Norepinephrine and serotonin release upon impact injury to rat spinal cord. J. Neurotrauma 7, 219–227 1707100 10.1089/neu.1990.7.219

[B107] Liu X. G. Sandkuhler J. (1995). Long-term potentiation of C-fiber-evoked potentials in the rat spinal dorsal horn is prevented by spinal N-methyl-D-aspartic acid receptor blockage. Neurosci. Lett. 191, 43–46 7659287 10.1016/0304-3940(95)11553-0

[B108] Liu Y. L. Zhou L. J. Hu N. W. Xu J. T. Wu C. Y. Zhang T. (2007). Tumor necrosis factor-alpha induces long-term potentiation of C-fiber evoked field potentials in spinal dorsal horn in rats with nerve injury: the role of NF-kappa B, JNK and p38 MAPK. Neuropharmacology 52, 708–715 10.1016/j.neuropharm.2006.09.011 17084420

[B109] Lovely R. G. Gregor R. J. Roy R. R. Edgerton V. R. (1986). Effects of training on the recovery of full-weight-bearing stepping in the adult spinal cat. Exp. Neurol. 92, 421–435 10.1016/0014-4886(86)90094-4 3956672

[B110] Lovely R. G. Gregor R. J. Roy R. R. Edgerton V. R. (1990). Weight-bearing hindlimb stepping in treadmill-exercised adult spinal cats. Brain Res. 514, 206–218 10.1016/0006-8993(90)91417-F 2357538

[B111] Ma J. Y. Zhao Z. Q. (2002). The involvement of glia in long-term plasticity in the spinal dorsal horn of the rat. Neuroreport 13, 1781–1784 12395122 10.1097/00001756-200210070-00017

[B112] Malinow R. Malenka R. C. (2002). AMPA receptor trafficking and synaptic plasticity. Annu. Rev. Neurosci. 25, 103–126 10.1146/annurev.neuro.25.112701.142758 12052905

[B113] Marino R. J. Ditunno J. F. Jr. Donovan W. H. Maynard F. Jr. (1999). Neurologic recovery after traumatic spinal cord injury: data from the Model Spinal Cord Injury Systems. Arch. Phys. Med. Rehabil. 80, 1391–1396 10569432 10.1016/s0003-9993(99)90249-6

[B114] McAllister A. K. Katz L. C. Lo D. C. (1999). Neurotrophins and synaptic plasticity. Annu. Rev. Neurosci. 22, 295–318 10.1146/annurev.neuro.22.1.295 10202541

[B115] McNaughton B. L. Barnes C. A. Rao G. Baldwin J. Rasmussen M. (1986). Long-term enhancement of hippocampal synaptic transmission and the acquisition of spatial information. J. Neurosci. 6, 563–571 3005525 10.1523/JNEUROSCI.06-02-00563.1986 PMC6568530

[B116] Mendell L. M. (1966). Physiological properties of unmyelinated fiber projection to the spinal cord. Exp. Neurol. 16, 316–332 10.1016/0014-4886(66)90068-9 5928985

[B117] Mendell L. M. Wall P. D. (1965). Responses of single dorsal cord cells to peripheral cutaneous unmyelinated fibres. Nature 206, 97–99 14334366 10.1038/206097a0

[B118] Mills C. D. Johnson K. M. Hulsebosch C. E. (2002). Group I metabotropic glutamate receptors in spinal cord injury: roles in neuroprotection and the development of chronic central pain. J. Neurotrauma 19, 23–42 10.1089/089771502753460213 11852976

[B119] Moser E. I. Krobert K. A. Moser M. B. Morris R. G. (1998). Impaired spatial learning after saturation of long-term potentiation. Science 281, 2038–2042 10.1126/science.281.5385.2038 9748165

[B120] Muller C. M. (1992). A role for glial cells in activity-dependent central nervous plasticity? Review and hypothesis. Int. Rev. Neurobiol. 34, 215–281 1587716 10.1016/s0074-7742(08)60099-9

[B121] Munro F. E. Fleetwood-Walker S. M. Mitchell R. (1994). Evidence for a role of protein kinase C in the sustained activation of rat dorsal horn neurons evoked by cutaneous mustard oil application. Neurosci. Lett. 170, 199–202 8058187 10.1016/0304-3940(94)90318-2

[B122] Ogoshi F. Yin H. Z. Kuppumbatti Y. Song B. Amindari S. Weiss J. H. (2005). Tumor necrosis-factor-alpha (TNF-[alpha]) induces rapid insertion of Ca2+-permeable [alpha]-amino-3-hydroxyl-5-methyl-4-isoxazole-propionate (AMPA)/kainate (Ca-A/K) channels in a subset of hippocampal pyramidal neurons. Exp. Neurol. 193, 384–393 10.1016/j.expneurol.2004.12.026 15869941

[B123] Patton B. C. Hook M. A. Ferguson A. R. Crown E. D. Grau J. W. (2004). The behavioral deficit observed following noncontingent shock in spinalized rats is prevented by the protein synthesis inhibitor cycloheximide. Behav. Neurosci. 118, 653–658 10.1037/0735-7044.118.3.653 15174945

[B124] Petruska J. C. Ichiyama R. M. Jindrich D. L. Crown E. D. Tansey K. E. Roy R. R. (2007). Changes in motoneuron properties and synaptic inputs related to step training after spinal cord transection in rats. J. Neurosci. 27, 4460–4471 10.1523/JNEUROSCI.2302-06.2007 17442831 PMC6672318

[B125] Prochazka A. Yakovenko S. (2007). Predictive and reactive tuning of the locomotor CPG. Integr. Comp. Biol. 47, 474–481 10.1093/icb/icm065 21672856

[B126] Prosser C. L. Hunter W. S. (1936). The extinction of startle responses and spinal reflexes in the white rat. Am. J. Physiol. 117, 609–618

[B127] Raineteau O. Schwab M. E. (2001). Plasticity of motor systems after incomplete spinal cord injury. Nat. Rev. Neurosci. 2, 263–273 10.1038/35067570 11283749

[B128] Roy R. R. Pierotti D. J. Baldwin K. M. Zhong H. Hodgson J. A. Edgerton V. R. (1998). Cyclical passive stretch influences the mechanical properties of the inactive cat soleus. Exp. Physiol. 83, 377–385 9639347 10.1113/expphysiol.1998.sp004121

[B129] Sandkuhler J. (2007). Understanding LTP in pain pathways. Mol. Pain 3, 9. 10.1186/1744-8069-3-9 17407590 PMC1852298

[B130] Sandkuhler J. Gruber-Schoffnegger D. (2012). Hyperalgesia by synaptic long-term potentiation (LTP): an update. Curr. Opin. Pharmacol. 12, 18–27 10.1016/j.coph.2011.10.018 22078436 PMC3315008

[B131] Sandkuhler J. Liu X. (1998). Induction of long-term potentiation at spinal synapses by noxious stimulation or nerve injury. Eur. J. Neurosci. 10, 2476–2480 10.1046/j.1460-9568.1998.00278.x 9749775

[B132] Shanthanelson M. Arvanian V. L. Mendell L. M. (2009). Input-specific plasticity of N-methyl-D-aspartate receptor-mediated synaptic responses in neonatal rat motoneurons. Eur. J. Neurosci. 29, 2125–2136 10.1111/j.1460-9568.2009.06769.x 19490018 PMC2931593

[B133] Sherrington C. S. (1906). Observations on the scratch-reflex in the spinal dog. J. Physiol. 34, 1–50 16992835 10.1113/jphysiol.1906.sp001139 PMC1465804

[B133b] Siddall P. J. (2009). Management of neuropathic pain following spinal cord injury: now and in the future. Spinal Cord 47, 352–359 10.1038/sc.2008.136 19002150

[B133a] Siddall P. J. McClelland J. M. Rutkowski S. B. Cousins M. J. (2003). A longitudinal study of the prevalence and characteristics of pain in the first 5 years following spinal cord injury. Pain 103, 249–257 12791431 10.1016/S0304-3959(02)00452-9

[B134] Stellwagen D. Beattie E. C. Seo J. Y. Malenka R. C. (2005). Differential regulation of AMPA receptor and GABA receptor trafficking by tumor necrosis factor-alpha. J. Neurosci. 25, 3219–3228 10.1523/JNEUROSCI.4486-04.2005 15788779 PMC6725093

[B135] Stellwagen D. Malenka R. C. (2006). Synaptic scaling mediated by glial TNF-alpha. Nature 440, 1054–1059 10.1038/nature04671 16547515

[B136] Stuck E. D. Christensen R. N. Huie J. R. Tovar C. A. Miller B. A. Nout Y. S. (2012). Tumor necrosis factor alpha mediates GABA(A) receptor trafficking to the plasma membrane of spinal cord neurons *in vivo*. Neural Plast. 2012, 261345 10.1155/2012/261345 22530155 PMC3317039

[B137] Thompson R. F. Spencer W. A. (1966). Habituation: a model phenomenon for the study of neuronal substrates of behavior. Psychol. Rev. 73, 16–43 5324565 10.1037/h0022681

[B138] Tillakaratne N. J. De Leon R. D. Hoang T. X. Roy R. R. Edgerton V. R. Tobin A. J. (2002). Use-dependent modulation of inhibitory capacity in the feline lumbar spinal cord. J. Neurosci. 22, 3130–3143 11943816 10.1523/JNEUROSCI.22-08-03130.2002 PMC6757541

[B139] Ugolini A. Corsi M. Bordi F. (1997). Potentiation of NMDA and AMPA responses by group I mGluR in spinal cord motoneurons. Neuropharmacology 36, 1047–1055 10.1016/S0028-3908(97)00103-2 9294969

[B140] van den Brand R. Heutschi J. Barraud Q. Digiovanna J. Bartholdi K. Huerlimann M. (2012). Restoring voluntary control of locomotion after paralyzing spinal cord injury. Science 336, 1182–1185 10.1126/science.1217416 22654062

[B141] Vichaya E. G. Baumbauer K. M. Carcoba L. M. Grau J. W. Meagher M. W. (2009). Spinal glia modulate both adaptive and pathological processes. Brain Behav. Immun. 23, 969–976 10.1016/j.bbi.2009.05.001 19435601 PMC2749915

[B142] Wang C. X. Nuttin B. Heremans H. Dom R. Gybels J. (1996). Production of tumor necrosis factor in spinal cord following traumatic injury in rats. J. Neuroimmunol. 69, 151–156 8823387 10.1016/0165-5728(96)00080-x

[B143] Washburn S. N. Patton B. C. Ferguson A. R. Hudson K. L. Grau J. W. (2007). Exposure to intermittent nociceptive stimulation under pentobarbital anesthesia disrupts spinal cord function in rats. Psychopharmacology 192, 243–252 10.1007/s00213-007-0707-1 17297638 PMC3222461

[B144] Wasner G. L. Brock J. A. (2008). Determinants of thermal pain thresholds in normal subjects. Clin. Neurophysiol. 119, 2389–2395 10.1016/j.clinph.2008.07.223 18778969

[B145] Watkins L. Martin D. Ulrich P. Tracey K. Maier S. (1997). Evidence for the involvement of spinal cord glia in subcutaneous formalin induced hyperalgesia in the rat. Pain 71, 225–235 10.1016/S0304-3959(97)03369-1 9231865

[B146] Watkins L. R. Drugan R. Hyson R. L. Moye T. B. Ryan S. M. Mayer D. J. (1984). Opiate and non-opiate analgesia induced by inescapable tail-shock: effects of dorsolateral funiculus lesions and decerebration. Brain Res. 291, 325–336 10.1016/0006-8993(84)91265-4 6697193

[B147] Weaver L. C. Marsh D. R. Gris D. Meakin S. O. Dekaban G. A. (2002). Central mechanisms for autonomic dysreflexia after spinal cord injury. Prog. Brain Res. 137, 83–95 12440361 10.1016/s0079-6123(02)37009-2

[B148] Whitlock J. R. Heynen A. J. Shuler M. G. Bear M. F. (2006). Learning induces long-term potentiation in the hippocampus. Science 313, 1093–1097 10.1126/science.1128134 16931756

[B149] Woolf C. J. (1983). Evidence for a central component of post-injury pain hypersensitivity. Nature 306, 686–688 6656869 10.1038/306686a0

[B150] Woolf C. J. Costigan M. (1999). Transcriptional and posttranslational plasticity and the generation of inflammatory pain. Proc. Natl. Acad. Sci. U.S.A. 96, 7723–7730 10.1073/pnas.96.14.7723 10393888 PMC33609

[B151] Woolf C. J. Salter M. W. (2000). Neuronal plasticity: increasing the gain in pain. Science 288, 1765–1768 10.1126/science.288.5472.1765 10846153

[B153] Woolf C. J. Thompson S. W. (1991). The induction and maintenance of central sensitization is dependent on N-methyl-D-aspartic acid receptor activation; implications for the treatment of post-injury pain hypersensitivity states. Pain 44, 293–299 1828878 10.1016/0304-3959(91)90100-C

[B154] Xiao M. Y. Zhou Q. Nicoll R. A. (2001). Metabotropic glutamate receptor activation causes a rapid redistribution of AMPA receptors. Neuropharmacology 41, 664–671 10.1016/S0028-3908(01)00134-4 11640920

[B155] Yezierski R. P. (1996). Pain following spinal cord injury: the clinical problem and experimental studies. Pain 68, 185–194 9121805 10.1016/s0304-3959(96)03178-8

[B155a] Yezierski R. P. (2000). Pain following spinal cord injury: pathophysiology and central mechanisms. Prog. Brain Res. 129, 429–449 10.1016/S0079-6123(00)29033-X 11098709

[B156] Yezierski R. P. (2009). Spinal cord injury pain: spinal and supraspinal mechanisms. J. Rehabil. Res. Dev. 46, 95–107 10.1682/JRRD.2008.06.0074 19533523

[B157] Young M. R. Fleetwood-Walker S. M. Mitchell R. Dickinson T. (1995). The involvement of metabotropic glutamate receptors and their intracellular signalling pathways in sustained nociceptive transmission in rat dorsal horn neurons. Neuropharmacology 34, 1033–1041 10.1016/0028-3908(95)00071-D 8532152

[B158] Zhang A. L. Hao J. X. Seiger A. Xu X. J. Wiesenfeld-Hallin Z. Grant G. (1994). Decreased GABA immunoreactivity in spinal cord dorsal horn neurons after transient spinal cord ischemia in the rat. Brain Res. 656, 187–190 10.1016/0006-8993(94)91383-8 7804836

[B159] Zhang G. H. Yoon Y. W. Lee K. S. Min S. S. Hong S. K. Park J. Y. (2003). The glutamatergic N-methyl–aspartate and non-N-methyl–aspartate receptors in the joint contribute to the induction, but not maintenance, of arthritic pain in rats. Neurosci. Lett. 351, 177–180 10.1016/j.neulet.2003.08.009 14623135

